# Stereotypical Processing of Emotional Faces: Perceptual and Decisional Components

**DOI:** 10.3389/fpsyg.2021.733432

**Published:** 2021-10-27

**Authors:** Daniel Fitousi

**Affiliations:** Department of Psychology, Ariel University, Ariel, Israel

**Keywords:** face perception, gender, emotion, General Recognition Theory, Garner's speeded classification paradigm, stereotypes, social cognition, social bias

## Abstract

People tend to associate anger with male faces and happiness or surprise with female faces. This angry-men-happy-women bias has been ascribed to either top-down (e.g., well-learned stereotypes) or bottom-up (e.g., shared morphological cues) processes. The dissociation between these two theoretical alternatives has proved challenging. The current effort addresses this challenge by harnessing two complementary metatheoretical approaches to dimensional interaction: Garner's logic of inferring informational structure and General Recognition Theory—a multidimensional extension of signal detection theory. Conjoint application of these two rigorous methodologies afforded us to: (a) uncover the internal representations that generate the angry-men-happy-women phenomenon, (b) disentangle varieties of perceptual (bottom-up) and decisional (top-down) sources of interaction, and (c) relate operational and theoretical meanings of dimensional independence. The results show that the dimensional interaction between emotion and gender is generated by varieties of perceptual and decisional biases. These outcomes document the involvement of both bottom-up (e.g., shared morphological structures) and top-down (stereotypes) factors in social perception.

## 1. Introduction

Close your eyes and try to imagine an angry face. What is the gender of this face? Now, imagine a happy or surprised face. What is the gender this time? It is likely that the first face was Male and the second was Female. People tend to associate angry expression with masculine faces, and happy or surprised expressions with feminine faces (Le Gal and Bruce, 2002; Atkinson et al., 2005; Becker et al., 2007). This bias has been dubbed: “the confounded nature of angry men and happy women” (Becker et al., 2007, p.179). However, the empirical evidence for a full fledged dimensional interaction between facial emotion and gender is divided. Le Gal and Bruce (2002) have applied Garner's speeded classification task (Garner, 1974b) to these dimensions and found that observers could attend selectively to facial emotion, while ignoring irrelevant variation on gender, and vice versa, a result that is inconsistent with the angry-men-happy-women confound. So, are the dimensions of facial emotion and gender integral or separable? And if the dimensions interact, how a bias is generated? Is it created at a sensory level? This might be possible because the same features that signal emotion are used by observers to decipher gender (Becker et al., 2007). Yet, the bias may emerge at a decisional level, since long-term learned or imagined associations (e.g., stereotypes) can potentially distort social judgment (Greenwald et al., 1998; Macrae and Bodenhausen, 2001; Quinn and Macrae, 2005; Freeman and Ambady, 2011). To answer these questions one needs to disentangle perceptual (low-level) and decisional (high-level) sources of interaction in the perception of facial emotion and gender. This is exactly what the current study aims to accomplish.

The current effort harnessed two complementary metatheoretical approaches in quest of resolution: Garner's logic of inferring informational structure (Garner, 1974b) and General Recognition Theory (GRT, Ashby and Townsend, 1986), which is a multidimensional extension of signal detection theory (Green and Swets, 1966; Ashby and Soto, 2015). The goals of the current study were to: (a) uncover the internal representation that govern the perception of gender and emotion, (b) disentangle various perceptual and decisional sources of dimensional interaction, and (c) relate operational and theoretical meanings of dimensional independence of emotion and gender. The outcome of this study offers a closer and detailed look at the intricate relations between facial emotion and gender. Most importantly, the study demonstrates the pervasive involvement of both perceptual and decisional biases in social perception.

## 2. The Angry-Men-Happy-Women Bias

Becker et al. (2007) have administrated various tasks to evaluate the relations between facial gender and emotion. The tasks spanned from open questionnaires, to speeded classification of gender or emotion, to an implicit association test (IAT, see for Greenwald et al., 1998), to rating of parametrically varied computerized faces. Outcomes from virtually all of these experimental procedures revealed that anger was associated with male faces and happiness with female faces. So, for example, in the speeded classification tasks, faces of angry men and happy women were categorized faster than faces of happy men and angry women. Several theoretical frameworks have been proposed to account for the angry-men-happy-women bias. According to the *ecological theory* (Gibson, 1979) perceptual features are associated with common actions. Thus, behavioral tendencies toward approach or avoidance, which are adaptive and should increase or decrease the organism's survival chances, are also related to perceptual dimensions. Consequently, perceptible features such as anger and masculinity are *affodances* that signal potentially dangerous objects from which one should avoid, whereas happiness, surprise, or femininity are affordances that signal potentially positive objects leading to approach. The ecological view predicts slower detection of negative stimuli, such as angry men, than positive stimuli such as happy women. The former are guided by avoidance, while the latter by approach. The direction of this prediction is opposite to the empirical findings (Le Gal and Bruce, 2002; Becker et al., 2007). But the ecological approach remains an important perspective in understanding the conceptual issues involved.

Another important framework that has been often put forward to account for biases is the *social learning* approach (Allport et al., 1954; Bodenhausen and Macrae, 1998; Macrae and Bodenhausen, 2001; Quinn and Macrae, 2005; Cloutier et al., 2014). According to this view, top-down processes are shaped by social stereotypes or real-world statistical associations. These mechanisms are similar to those studied in category prototypicality effects (Van Orden, 1987), semantic priming (Schvaneveldt and McDonald, 1981), or the word frequency effect (Glanzer and Bowles, 1976). The associationist approach correctly predicts better recognition of happy female and angry male faces than angry females and happy males faces. Men more often than women display violent and threatening behavior (Trivers, 1985), whereas women are more friendly and peaceful (Taylor et al., 2000). Women smile more than men (LaFrance et al., 2003) and men express anger more frequently (Fabes and Martin, 1991). These prior statistical regularities may affect the way people decide about facial emotion and gender by inducing top-down strategies. An alteration in the observer's response bias is therefore a candidate mechanism for bias in social perception.

Finally, a bottom-up approach (Freeman and Ambady, 2011; Johnson et al., 2012) postulates that the morphological structures signaling emotion and gender are not independent from each other. Hence, the cues that observers take advantage of to decipher facial expression and gender are confounded (Becker et al., 2007). Darwin (1872) and Ekman (1976) noted that anger is conveyed by several cues such as flared nostrils, constricted mouth, and “flashing eyes” created by retraction of the eyelids. These cues are also characteristics of masculine faces: men have larger brows, more angular jaws, thinner lips relative to women, and larger noses which make the nostrils look flared. Women, on the other hand, have rounder features, higher brow-to-lid distance, smaller noses, and fuller lips. Along these lines, Le Gal and Bruce (2002) predicted that: “Male faces especially should appear less masculine when surprised, since the increased brow-to-lid distance is a cue associated with female faces. Female faces should look less feminine when angry, since the reduced brow-to-lid distance is characteristic of male faces.” (p.234). The upshot is that a bias in social categorization may reflect a bias in perception not decision^1^.

## 3. Emotion and Gender: Integral or Separable Dimensions?

The case for emotion-gender interaction seems strong, but it turns out that not all canonical face recognition models predict it. Bruce and Young (1986)'s dual-route model has been the dominant approach to face recognition over the last 40 years or so. The model famously argues for a functional and representational independence between information processing routes for derivation of different information from faces. For example, the model holds that identity recognition relies on invariant, long-term, abstract representations, stored in face-recognition-units (FRUs), that are separate and independent from representations of other aspects of faces. This prediction has been supported by a wealth of neurological (Humphreys et al., 1993; Hornak et al., 1996; Roudier et al., 1998), computational (Calder et al., 2001), and imaging (Breiter et al., 1996; George et al., 1999) studies (but see Fitousi and Wenger, 2013). With respect to facial gender and emotion, the dual-model's predictions are not as clear cut. Gender is indeed an invariant dimension of faces like identity, and therefore may or may not be processed along a dedicated route. Summarizing a large body of work, Le Gal and Bruce (2002) concluded that while there is evidence that both gender and emotion are independent from identity, no study offers a direct test of their independence.

Le Gal and Bruce (2002) entertained two apparently contrasting hypotheses: (a) gender and emotion are independent in processing, because they are governed by separate processing routes as predicted by the dual-route model, and (b) gender and emotion interact because discrimination of values on the two dimensions rely on variations on the same feature—brow-to-lid distance. Thus, raised eyebrows indicate the face is female, and lowered eyebrows indicates the face is angry, but also that the face is male. To test the these hypotheses Le Gal and Bruce (2002) have used speeded and non-speeded methodologies. In a rating task, participants assessed the masculinity of male and female angry and surprised faces. The results revealed that participants rated surprised faces as more feminine than angry faces, an outcome that is inconsistent with the representational independence postulated by the dual-route model. In a Garner's speeded classification task participants categorized faces on emotion and ignored variations on gender. A detailed explanation of the Garner paradigm is presented in the next section, but for now it should be noted that the Garner paradigm is comprised of two primary blocks, a baseline block, in which the irrelevant dimension (gender) is fixed at one value (e.g., female), and a filtering block, in which relevant and irrelevant values vary in an orthogonal fashion. If performance in filtering is worse than in baseline, than Garner interference is recorded, and the dimensions are considered as integral or dependent (Garner, 1974b). The results in this task were somewhat opposite to those observed in the rating task. Participants could attend selectively to either dimension without suffering interference from irrelevant variation on the other, a result suggesting independence. However, when the authors looked at the stimulus level within the Garner blocks, they found that participants responded faster to angry-men and surprised-women faces than to angry-women and surprised-men faces, a result suggesting an interaction. A subsequent Garner study by Atkinson et al. (2005) replicated the stimulus level interaction, and found the dimensions to be only partially independent in the Garner task. Classification of gender were not affected by irrelevant variation on emotion, but judgments of emotion were interfered by irrelevant variation on gender. Taken together, the outcomes from the studies by Le Gal and Bruce (2002) and Atkinson et al. (2005) pose a conundrum. Are emotion and gender independent or dependent in processing?

Possibly unaware of the preceding Garner results, Becker et al. (2007) have argued conclusively that: “perceptual dimensions of gender and anger or happiness are …not separable from each other (Garner, 1974b; Ashby and Townsend, 1986)” (p. 189). Curiously enough, these authors cite the two major exponents of GRT (Ashby and Townsend, 1986) and Garner (Garner, 1974b) paradigms, but without actually deploying their methodologies, nor consulting earlier studies that had done so. In the context of the Garner and GRT frameworks, it is apparent that the interaction captured by Becker et al. (2007) is only one facet of the possible types of dimensional relations. An interaction at the stimulus level can occur in tandem with separability at the dimensional level (Le Gal and Bruce, 2002; Atkinson et al., 2005). This point has been succinctly put by Le Gal and Bruce (2002): “…there is a distinction to be drawn between information processing operations that may be independent (or separable, in Garner's terms) and interactions at the level of stimulus pattern, which may affect the way an item is assigned to a category.” (p.242). It is this distinction and pertinent conceptual issues the current study addresses in a rigorous fashion.

## 4. The Current Study

The evidence reviewed so far strongly suggests that the notions of interaction (and its complementary independence) in processing and representation of emotion and gender should be systematically investigated. An important caveat to issue at this point, is the actual definition of independence (Garner and Morton, 1969; Fitousi, 2013; Fitousi and Wenger, 2013; Von Der Heide et al., 2018). In all of the studies to date, the definitions have been operational. Specifically, both the presence of the angry-men-happy-women bias and the absence of Garner interference rely on the computations of differences between mean RTs across conditions or blocks. This is problematic because: (a) such differences can be generated by several psychophysical factors that may or may not reflect genuine interaction between the dimensions (Garner and Morton, 1969; Fitousi, 2015; Algom and Fitousi, 2016), (b) some of the empirical measures did not converge and even if they did, (c) the theoretical concepts deduced from each task is only a restatement of the empirical result specific to that task, and finally (d) there are several ways by which independence of dimensions can be violated (or interaction to emerge), and the traditional measures deployed so far could not potentially disentangle them. Consequently, I chose to approach the issue using a theoretical characterization of independence–General Recognition Theory (GRT, Ashby and Townsend, 1986), along with the operational definitions which are central to the notion of separability in the Garner approach (Garner et al., 1956; Garner and Morton, 1969). The two-pronged attack should allow us to link the earlier Garner findings with the more theoretically-driven rigorous approach of GRT, and thus to offer a more rigorous account of the empirical phenomena.

### 4.1. The Garner Test: Are Emotion and Gender Separable or Integral Dimensions?

Garner argued for a fundamental partition between *integral* and *separable* dimensions that combine to create objects in our perception (Garner, 1962, 1970, 1974a,b, 1976, 1991). This distinction is a pillar of modern cognitive science (Algom and Fitousi, 2016). Objects made of integral dimensions, such as hue and saturation are perceived in their totality and cannot be decomposed easily and free of mental effort. Objects made of separable dimensions, such as shape and color, can be readily decomposed into their constituent dimensions. The *Garner interference* is a quantitative assay of the processing of integral dimensions. Are gender and emotion integral or separable?

There is an important caveat to issue at this point. The integral-separable distinction cannot be decided based only on the presence or absence of Garner interference. To avoid circular reasoning, Garner has noted the need for converging operations (Garner et al., 1956). In the absence of converging operation, any theoretical concept is only a restatement of the empirical result (Fitousi, 2015; Von Der Heide et al., 2018). Therefore, the integral-separable distinction is now supported by converging operations from similarity scaling (Attneave, 1950; Melara, 1992), information theory (Garner, 1962; Garner and Morton, 1969; Fitousi, 2013), and system factorial technology (Townsend and Nozawa, 1995). In addition, Ashby, Townsend and their colleagues developed the framework of general recognition theory (GRT). This approach provides further independent theoretically-driven definitions for the concept of separability and other related concepts (Maddox and Ashby, 1996; Townsend et al., 2012; Fitousi, 2013). The GRT is applied here along with the Garner paradigm with the same set of stimuli to (a) provide a set of converging operations and (b) expand the scope of investigation into theoretically-driven definitions of independence.

### 4.2. The Garner Paradigm

**Figure 1:**
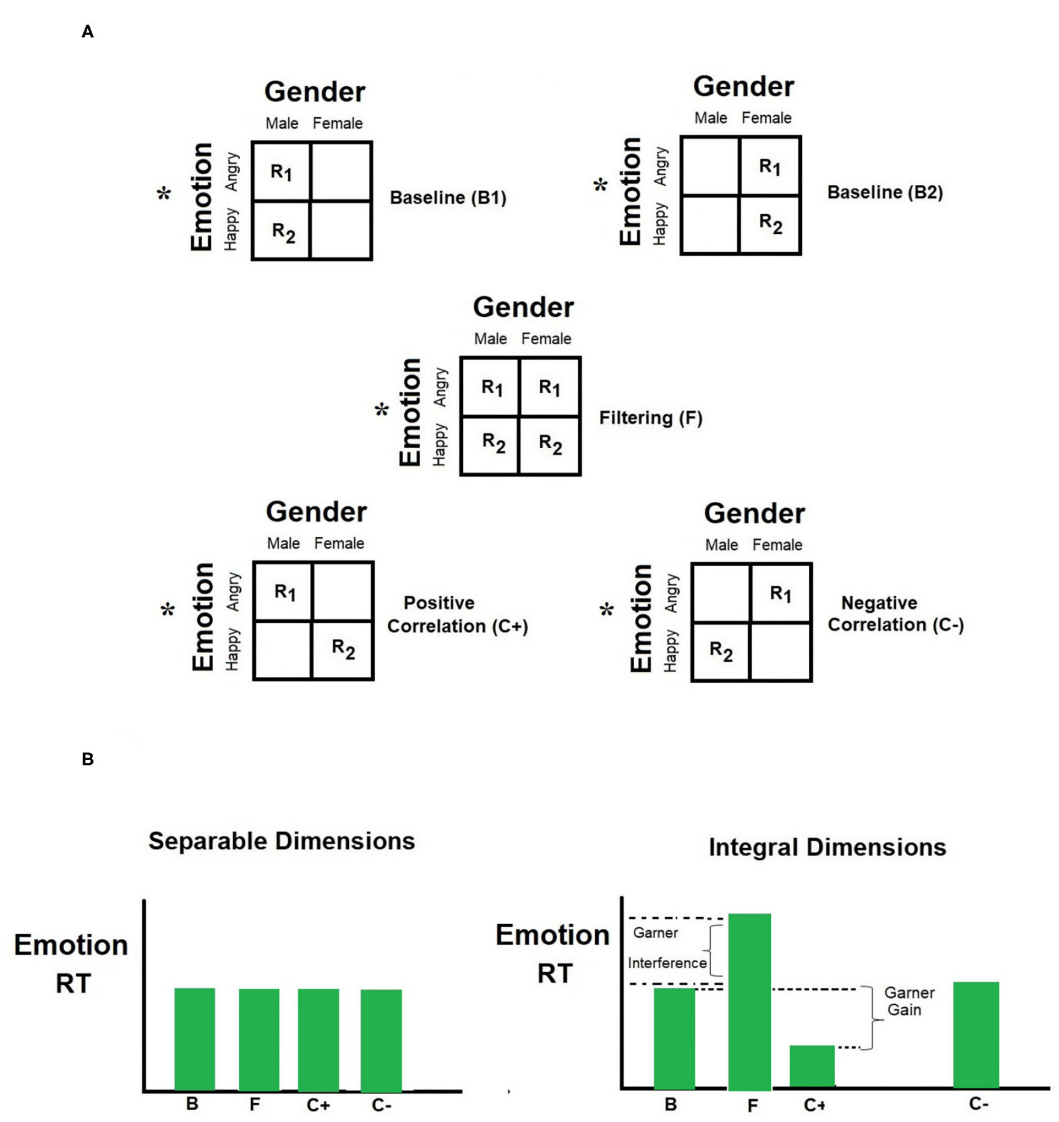
**(A)** Schematics of Garner's speeded classification paradigm used in Experiment 1 of this study. The three basic conditions–baseline (B), filtering (F), and correlation (C)—are depicted on the top panel. The asterisk indicates that facial emotion, not facial gender, was the relevant dimension for responding. R1 and R2 depict the correct responses to the two values of the relevant dimension. The correlation condition depicts two possible ways by which values from the relevant dimension co-varied with values from the irrelevant dimension. **(B)** Two prototypical outcomes, tapping separable and integral processing are illustrated at the bottom panel.

Consider the application of the paradigm to facial gender and emotion (see Figure 1). The design consists of three blocks. The observer's task is to decide, while timed, on the emotion of the face, while ignoring its gender. In the baseline block, the task-irrelevant gender was held constant at one value (e.g., male). In the filtering block, the task-irrelevant changed from trial-to-trial in a random fashion. In the correlation blocks, the task-irrelevant gender dimension changed in tandem with the target emotion dimension. In the positively correlated block, men faces were angry and women faces were happy; whereas in the negatively correlated block, men faces were happy and women faces were angry. The sign of the correlation is based on the predicted angry-men-happy-women bias (Becker et al., 2007).

The difference in mean RT between the baseline and filtering blocks defines the measure know as *Garner interference* (Pomerantz, 1983):


(1) $$\begin{array}{rcl} Garner\;Interference & = & MRT\;\left(filtering\right)\;-\;MRT\;\left(baseline\right) \end{array}$$

where MRT is the mean latency to classify the face on emotion (as angry or happy). An analog formula exists for error. The presence of a Garner interference implies that irrelevant variation on the gender of the face took a toll on performance with the emotion of the face, and that selective attention to the relevant dimension failed. In addition, the complete Garner paradigm consists of a third condition, correlation, where values of the irrelevant gender also varies from trial to trial. However, under this condition, they vary in correspondence with the values of emotion. Under positive correlation, the values of gender and emotion always match (men are angry and women happy); under negative correlation the values of the two dimension mismatch (men are happy and women angry). Performance in the correlation blocks is often better than that in baseline, an advantage that is dubbed *redundancy gain*.


(2) $$\begin{array}{rcl} Redundancy\;Gain & = & MRT\;\left(baseline\right)-MRT\;\left(correlated\right) \end{array}$$

This is because the irrelevant dimension is now predictive of the relevant dimension. When Garner design includes both positively and negatively correlated blocks, as in the current study, an across blocks Stroop-like effect can be measured as the difference between the negative and positive correlation blocks (see for, Melara and Mounts, 1993):


(3) $$\begin{array}{l} Stroop\;effect=MRT\;(negative\;correlation)\\ \;-\;MRT\;(positive\;correlation) \end{array}$$

Recall that in the present study, a positive correlation block consists of angry-men or happy women faces, whereas a negative blocks includes angry-women or happy men faces. The two blocks include compatible and incompatible stimuli. A difference in performance in the two blocks amounts to a Stroop-like effect (Algom and Fitousi, 2016). The effect entails that participants processed the semantic meaning of the irrelevant dimension.

Garner (1974a,b, 1976, 1991) based his definition of integral and separable dimensions on these primary measures. Integral dimensions are those that produce both Garner interference and redundancy gains. Therefore, the following RT inequalities define integral dimensions:


(4) $$\begin{array}{r} MRT\;\left(correlation\right)<MRT\;\left(baseline\right)<MRT\;\left(filtering\right) \end{array}$$

whereas separable dimensions produce no Garner interference or redundancy gains:


(5) $$\begin{array}{r} MRT\;\left(correlation\right)=MRT\;\left(baseline\right)=MRT\;\left(filtering\right) \end{array}$$

I deployed again the same three types of blocks of trials with gender as the target dimension and emotion as the irrelevant dimension. The RT equations apply to the resulting pattern of results. It should be noted at this point that given a pair of dimensions, A and B, A can be separable with respect to B while B is integral with A (Garner, 1974b; Fitousi and Algom, 2020). For example, Atkinson et al. (2005) found that gender is integral with emotion, but emotion is separable from gender. It should be also noted that although Garner interference and redundancy gains point to integral processing, they may not always surface together. There are cases when only one or some of these patterns are documented in the data (Fitousi, 2020). Specifically, redundancy gains have been ascribed to a decisional rather than a perceptual effect (Ashby and Maddox, 1994).

In sum, the Garner paradigm provides a powerful means of assessing perceptual interaction. However, its definitions are operational, it does not separate perceptual from decisional interactions, and requires additional converging operations from related methodologies. One such methodology is the general recognition theory (Ashby and Townsend, 1986).

## 5. General Recognition Theory Interrogation of Facial Emotion and Gender

General recognition theory (GRT, Ashby and Townsend, 1986) is a multidimensional elaboration of signal detection theory (SDT, Green and Swets, 1966). It is augmented by surrogate methodologies and statistical packages (Townsend et al., 1981, 2012; Kadlec and Townsend, 1992; Kadlec, 1995; Thomas, 2001; Silbert and Thomas, 2013, 2014; Soto et al., 2015, 2017; Fitousi, 2018). It allows researchers testing hypotheses regarding the independence of dimensions at both the dimension and stimulus level (Fitousi and Wenger, 2013; Fitousi, 2014). The GRT is applied to factorial designs in which stimuli are generated by intersecting the values of two dimensions. In the current experiments, two levels of gender (man, woman) were intersected with two levels of emotion (angry, happy) to create four face categories (i.e., angry man, angry woman, happy man, happy woman).

On each trial, a single face is presented. The task of the observer is to identify the levels of both gender and emotion by producing two responses—one indicating the gender of the face (man or woman?) and the second indicating the emotion of the face (angry or happy?). The task is non-speeded, and performance is kept sub-optimal to the effect that observers commit identification errors. The critical data are summarized in an identification confusion matrix in which stimulus-response frequencies are cross-tabulated (see Townsend et al., 1981). Table 1 is an example of such a matrix. The diagonal entries give the frequency of trials in which both gender and emotion were identified correctly, whereas the off-diagonal entries give the frequency of trials in which observers erred in identifying one or two of the dimensions. The GRT accounts for the pattern of confusion by making inferences regarding the internal representations of the stimuli, their configuration, and the decision rules employed by the observer (Ashby and Townsend, 1986).

**Table 1:** Exemplary identification confusion matrices for GRT.

**Stimulus**	**Angry/Male**	**Angry/female**	**Happy/Male**	**Happy/Female**
**Response**				
Angry/Male	140	36	34	40
Angry/Female	89	91	4	66
Happy/Male	85	5	90	70
Happy/Female	20	59	8	163

**Figure 2:**
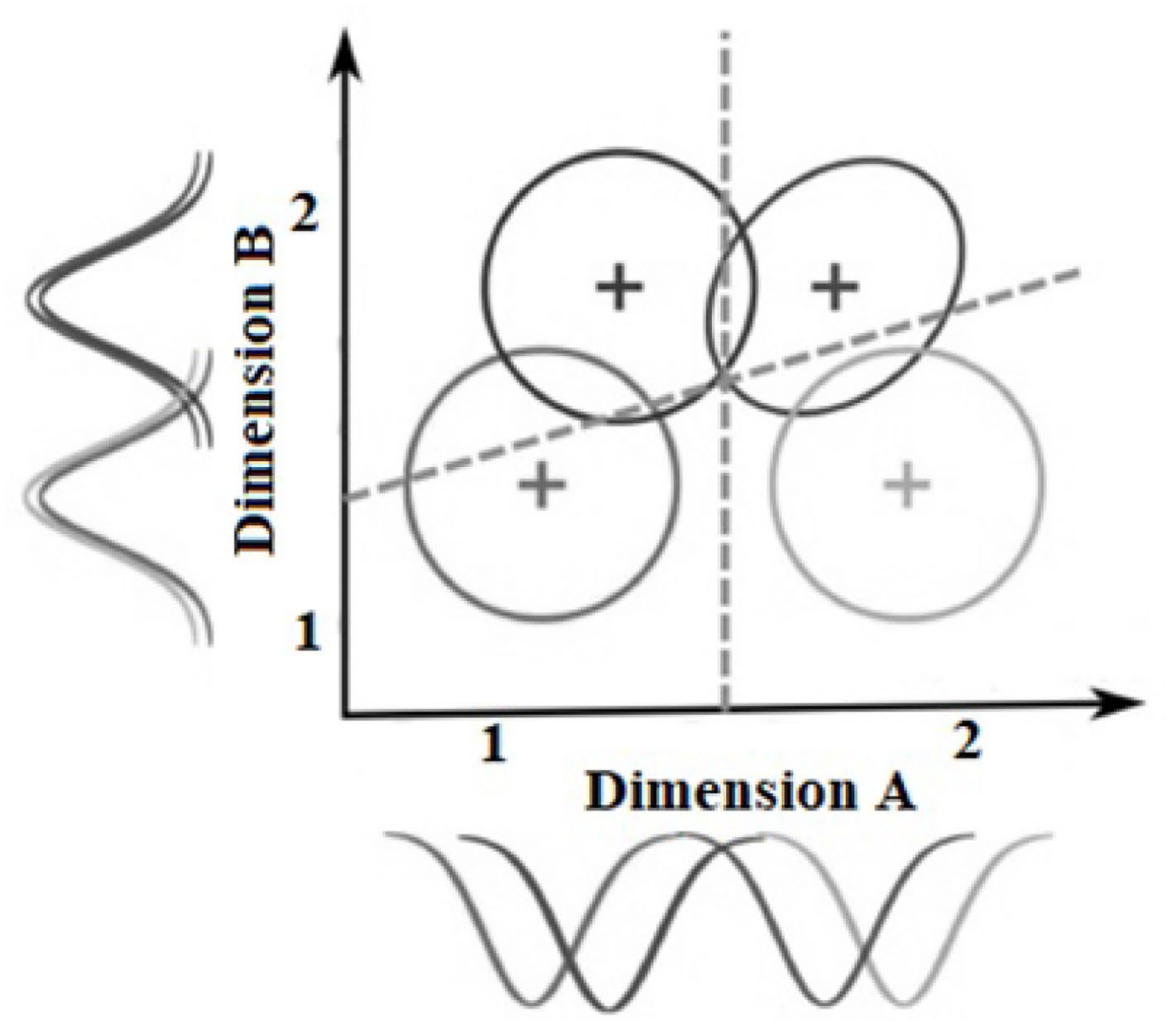
An example of a General Recognition Theory space with two dimensions A and B that vary on two levels 1 and 2. Each circle/ellipse represents the perceptual effect of one stimulus. The dashed lines represent the decision bounds, and the distributions on the *x*-axis and *y*-axis represents the unidimensional marginal distributions that in combination create the bidimensional distributions.

GRT relies on the premise that each stimulus elicits a unique perceptual effect in the observer (Green and Swets, 1966). The perceptual effect varies in strength across trials and can be best represented as a multidimensional distribution comprised of a mean and covariance matrix in a Cartesian space (Ashby and Townsend, 1986; Ashby and Perrin, 1988; Ashby and Maddox, 1994)^2^. In the factorial design, this reflects the combined perceptual effects of two marginal distributions—one for each dimension (see Figure 2). Another fundamental assumption is that the observer partitions the psychological space into four mutually exclusive response regions by placing two decision bound along the *x* and *y* axes, one per each dimension. These boundaries assist the observer to decide from which of the four stimuli has the perceptual effect arrived. The interplay between the four multidimensional distributions and the decision bounds determine the pattern of confusion among stimuli. When the four multidimensional distributions are cut horizontally, they create equal-likelihood contours (see Figure 2).

**Figure 3:**
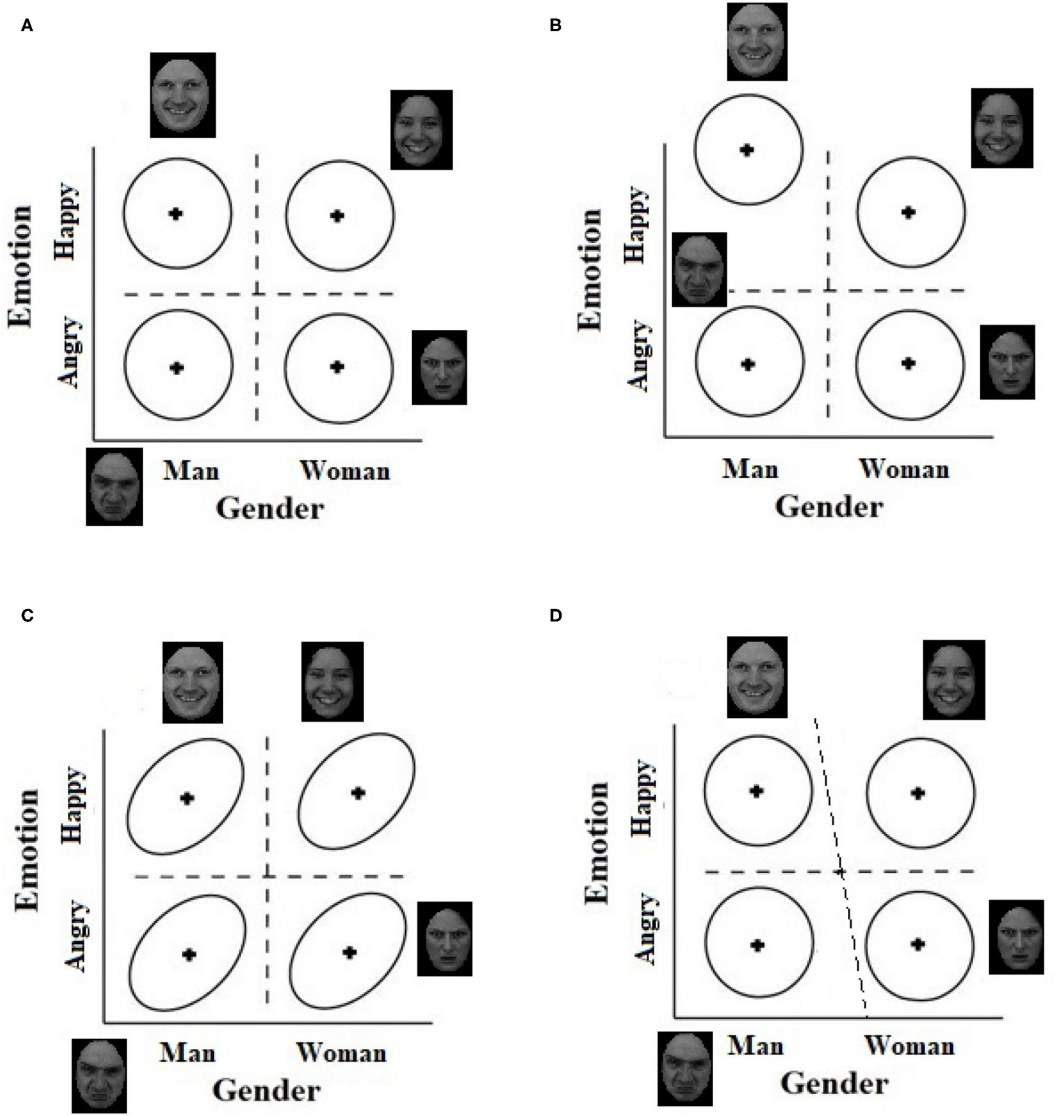
General recognition theory (GRT) spaces in which: **(A)** Perceptual independence, perceptual separability, and decisional separability hold, **(B)** Perceptual separability is violated on the emotion dimension, **(C)** Perceptual independence is violated in all stimuli, and **(D)** Decisional separability is violated on the gender dimension.

A main assumption in GRT is the existence of three types of independence: (a) *perceptual independence*, (b) *perceptual separability*, and (c) *decisional separability* (Ashby and Townsend, 1986). Any one of these constructs can be violated by the observer. These violations are being tested in the current study to diagnose the source of bias (if any) in judgments of emotion and gender. Perceptual independence refers to a form of within-stimulus statistical independence that holds if and only if the perceptual effects of the components that comprise the stimulus (e.g., male, angry) are stochastically independent. When perceptual independence holds in a stimulus, the shape of equal-likelihood contour is a circle (Figure 3A). However, when perceptual independence is violated, the two marginal distributions are correlated, and the equal-likelihood contour takes the shape of a tilted ellipse (Figure 3C).

Perceptual Separability holds if the discriminability of one dimension remains constant over levels of the other dimension (Fitousi and Wenger, 2013; Fitousi, 2017a). For example, in Figure 3A, the distance between the means of the multidimensional distributions for “angry man” and “happy man” are the same as for “angry woman” and “happy woman.” When perceptual separability holds for both dimensions, the means of the multidimensional distributions should form a rectangle. However, when perceptual separability is violated on one of the dimensions, say emotion, the discriminability of the dimension varies as a function of the other dimensions, say gender. In Figure 3B, for example, the observer finds it easier to discriminate between angry and happy men faces than between angry and happy women faces. As a result, the configuration changes into a trapezoid. Of course, perceptual separability can be also violated on both dimensions.

Decisional Separability holds if and only if the decision bounds placed by the observer are orthogonal to the axes and to each other (Maddox, 1992). In Figure 3A, the decision bound for categorizing gender (i.e., man or woman?) remains constant whether the facial emotion is angry or happy, and we can say that gender is decisionaly separable from emotion, and in this example, the same is true for emotion with respect to gender. Violations of decisional separability on one of the dimensions occurs when the decision bound for that dimension in not orthogonal to that of the other dimension (Maddox, 1992). In Figure 3D, decisional separability is violated on the gender dimension because the decision bound for facial gender is different for angry and for happy faces. For angry faces the bound is closer to women faces, but for happy faces it is closer to men faces. Such a violation can generate the angry-man-happy-woman confound.

Note that perceptual and decisional separability are across-stimulus constructs because their violations relies on the relations between the representations of the four face categories. Perceptual independence, in contrast, is a within-stimulus concept, and it can hold or fail separately in each of the four individual stimuli. This distinction is important for studying social bias in judgment of faces because it implies that a pair of dimensions can be perceptually and decisionally separable (Le Gal and Bruce, 2002), but still demonstrate failures of perceptual independence within an individual face category (Fitousi, 2014, 2018).

A comment is in order regarding the relations between the Garner and GRT paradigms. The two methodologies have been developed to assess independence of dimensions. A question that often arises is whether the notions of perceptual and decisional separability in GRT are related to the notions of separability/integrality and redundancy gain in the Garner's paradigm (Fitousi and Wenger, 2013). Theoretically, the answer is yes. Ashby and Maddox (1994) have developed a response time model that relates GRT spaces to Garnerian concepts using a random-walk model (see also Maddox, 1992). These authors showed that Garner interference is compatible with violations of perceptual separability in GRT, whereas Garner's redundancy gain (measured with the correlated blocks) is compatible with violations of decisional separability. However, this maxim has not stood well under empirical testing. For example Fitousi and Wenger (2013) have shown that Garner interference can be accompanied by violations of both perceptual and decisional separability in GRT. One of the goals of the present study has been to assess the degree to which the Garner results with emotion and gender (Le Gal and Bruce, 2002; Atkinson et al., 2005) correspond with the outcomes from the GRT paradigm.

## 6. The Experiments

Three experiments were conducted. Experiment 1 was designed to test the claims made by Becker et al. (2007), that gender and emotion are integral dimensions. Experiment 1 used the exact intersection of levels deployed in that study (i.e., angry/happy and men/women). This also allowed a conceptual replication of Le Gal and Bruce (2002)' results, whose faces incorporated a slightly different intersection of levels (i.e., angry/surprised and men/women). Experiment 1 was also designed to validate the face stimuli, and set the stage for the application of the GRT methodology. In Experiment 2, the same stimuli from Experiment 1 were subjected to the (non-speeded) GRT methodology. The goal here was to assess the role of perceptual and decisional biases and also relate the outcomes to those from the Garner's test. In Experiment 3, a new set of faces with the angry/surprised and men/women intersections was tested in the GRT methodology. Experiment 3 served as a replication and extension of Experiment 2, and it also provided the link to the Garner results adduced by Le Gal and Bruce (2002) with the same intersections of levels.

The logic guiding the current study is that the underlying psychological representation remains the same irrespective of the measurement operations, (Garner vs. GRT), exposure times, dependent variables, and other differences in experimental methods. This logic should hold if the conceptual structures gauged by these operations are related. This approach is well known as the *converging operation method* (Garner et al., 1956; Von Der Heide et al., 2018). A similar approach has been advanced by Becker et al. (2007), who have harnessed several approaches (RTs, accuracy, rating tasks) to study the relations between gender and emotion and found converging evidence for the interaction between gender and emotion.

The central hypothesis advanced in the present study concerns the involvement of both perceptual and decisional sources in the categorization of facial emotion and gender. The goal of the current work is therefore to dissociate between these two sources. In addition, I offer a related corollary assuming close correspondence between perceptual interactions and bottom-up factors (e.g., structural interactions among features), and between decisional interactions and top-down factors (e.g., stereotypes, goals). This dichotomy has been criticized in the area of visual attention (Awh et al., 2012), but it still enjoys support from prominent researchers (Theeuwes, 2010). The dichotomy has not stopped playing a central theoretical role in many areas of social cognition (Freeman and Ambady, 2011), categorization (Ashby and Soto, 2015) and face recognition (Wegner and Ingvalson, 2002)^3^. The proposed mapping is of broad theoretical generality, and as such does not rely on a specific factor/s. It only serves as a plausible candidate mechanism to account for the dissociation between perceptual and decisional effects.

## 7. Experiment 1

### 7.1. Method

#### 7.1.1. Participants

Twenty eight young students from Ariel University took part (F = 24, M = 4, mean age = 23.34). All had normal or corrected-to-normal vision. All gave their signed consent. Becker et al. (2007) reported that the angry-man-happy-woman interaction is characterized by effect sizes of 0.49 (for RTs) and 0.56 (for accuracy). I have used the “pwr” package (Champely et al., 2018) in the statistical software R (R Core Team, 2017) to compute the sample sizes. The design was a 2 x 2 ANOVA with repeated measures. There were two factors (Emotion, Gender) and two levels (Angry vs. Happy, and Male vs. Female). The power was set to 0.80 and the significance level was set to 0.05 for these effect sizes. The N required were found to be 13. So, the sample sizes used here are at least double than is required.

### 7.2. Stimuli and Apparatus

**Figure 4:**
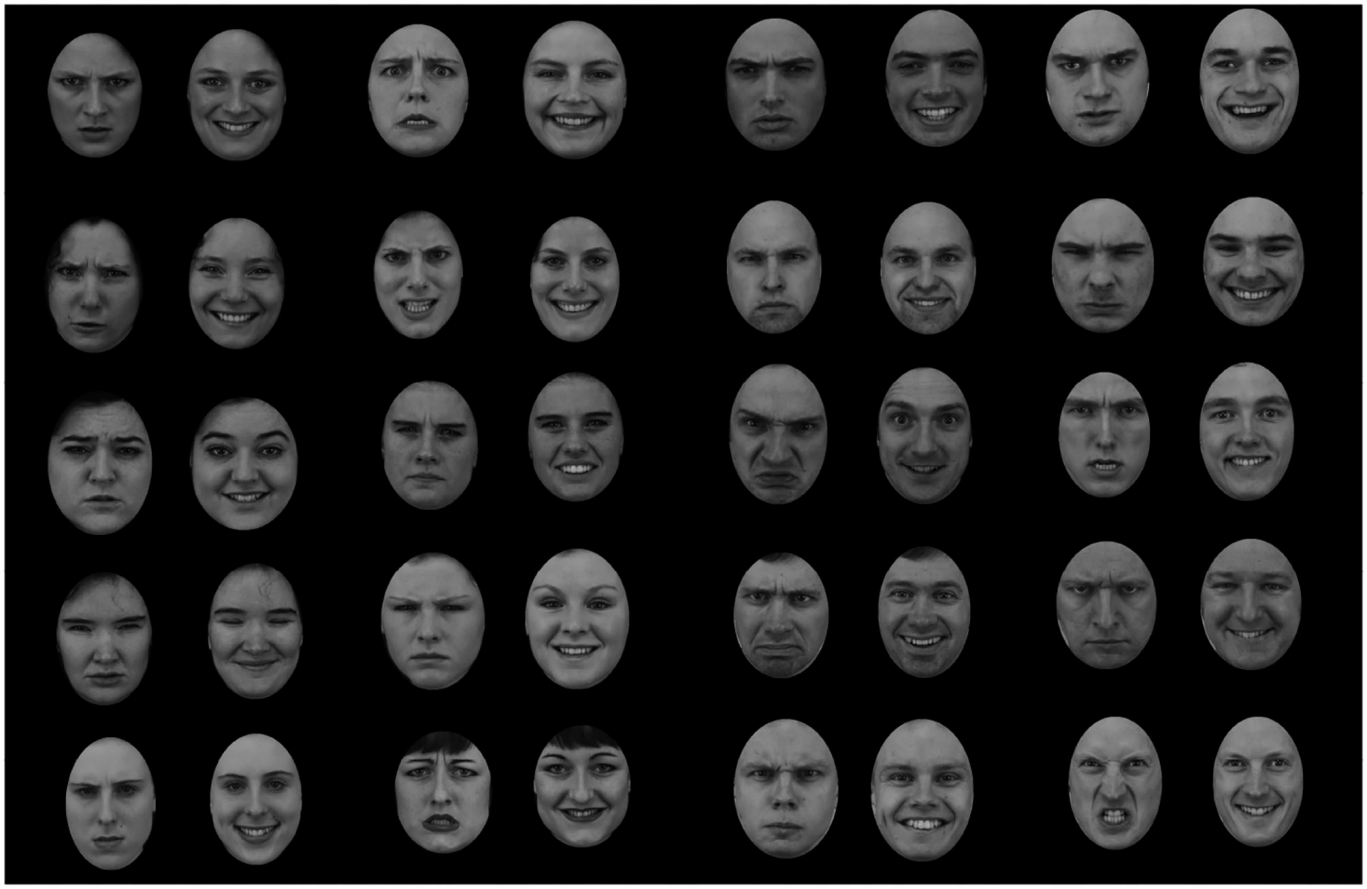
The face stimuli presented in Experiment 1 and Experiment 2. The faces were created by intersecting two levels of emotion (angry and happy) and two levels of gender (male, female). The images were taken with permission from the Karolinska Directed Emotional Face (KDEF, Lundqvist et al., 1998).

The face images were taken with permission from the Karolinska Directed Emotional Face (KDEF, Lundqvist et al., 1998). The free GIMP software was used to standardize all the faces by cropping the hair of each face and placing it within a standard oval shape of approximately 7 × 9 cm. Viewed from an approximate distance of 56 cm the stimuli subtended 9.09° × 7.12° visual angle. The faces were generated by a factorial intersection of the two dimensions, yielding four types of faces: angry man, angry woman, happy man, and happy woman. The same facial identity could appear in either angry or happy expression. 20 different identities (10 females and 10 males) were recruited from the KDEF archive. From these identities I created 10 faces for each combination of gender and emotion. This resulted in 40 faces in total (see Figure 4). The faces in the KDEF archive are standardized with respect to photographic aspects (e.g., pose, lightning). In addition, all faces were of the same ethnicity (Caucasian). To the best of my knowledge, the faces in the KDEF archive are not controlled for dominance. The experiment was programmed with Adobe Macromedia Authoware software (Macromedia, 1987).

## 8. Procedure and Design

Participants were sitting in front of a laptop computer in a dimly lit room. On each trial a single face was randomly chosen by the computer and presented at the center of the screen until response. It was replaced by a blank screen for 300 ms and then a new face was presented. Each participant performed in two tasks: (a) classification of gender, and (b) categorization of emotion. Each task consisted of 5 blocks (two baselines, two correlations, and one filtering). Baseline or correlated blocks included 20 trials, and filtering block included 40. The four basic conditions were: baseline (B), filtering (F), positive correlation (C+) and negative correlation (C−). A complete cycle of all 5 Garner blocks consisted of 120 trials in a given task. Each task cycle was performed three times in a random order. The order of tasks was chosen randomly by the computer, such that half of the participants started with the gender task and half with the emotion task. The blocks associated with each task were performed together. The order of blocks within a task was randomized. In this procedure participants switch between the tasks only once, so effects of carryover from one task to the other are minimal. Moreover, a 2-min break separated each block. This guarantees that each block and each task are minimally affected by the previous ones (Garner and Felfoldy, 1970; Algom et al., 1996; Algom and Fitousi, 2016). This notion has also been supported by statistical analysis. Overall, each participant completed 720 trials. Participants responded with either their right or left hand by pressing the button keys (“m” or “z”).

In the gender task participants classified the faces along the gender dimension. In the filtering block, gender and emotion varied orthogonally and all combinations of gender (male, female) and emotion (angry, happy) were presented randomly. In the baselines blocks gender varied from trial-to-trial, but emotion was held fix at one value (e.g., angry). There were two baseline blocks: in one baseline the irrelevant dimension (emotion) was restricted to angry faces, and in the second baseline it was restricted to happy faces. In the correlated blocks, gender covaried with emotion. There were two correlated blocks. These blocks reflected either positive or negative correlations of the dimensional values depending on the social bias they conveyed. A positively correlated block (C+), was one in which only angry-man and happy-woman were presented. A negatively correlated block (C−), was one in which only angry-woman and happy-man faces were presented. The structure of the emotion task was comparable, but with gender as the irrelevant dimension.

## 9. Results

**Figure 5:**
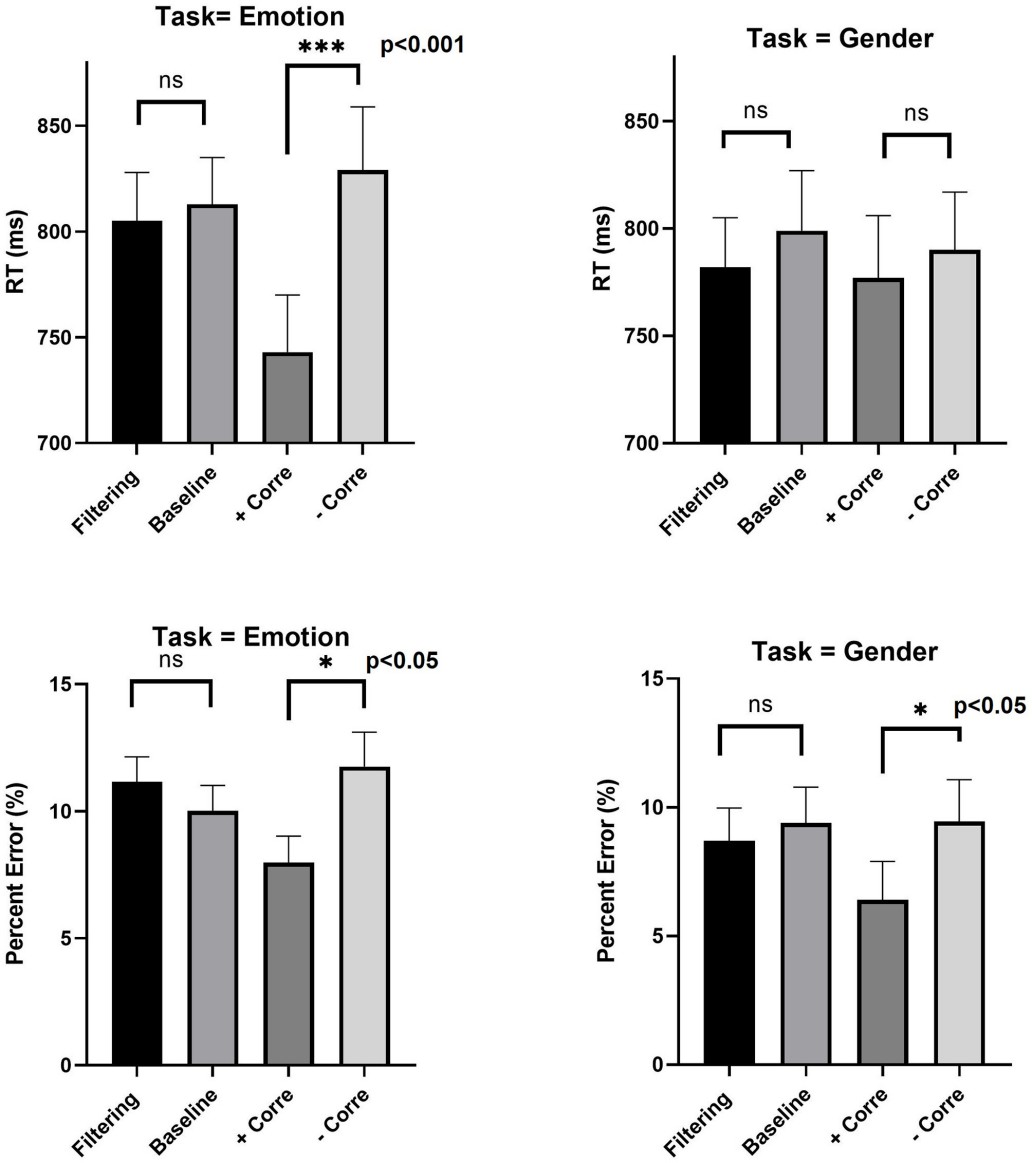
Experiment 1: Mean RTs and percent error in filtering, baseline, positive correlation and negative correlation for emotion and gender tasks. Error bars are standard error of the mean. ***p < 0.001, **p < 0.01, and *p < 0.05.

Data can be downloaded from https://data.mendeley.com/datasets/zrz7krjhr4/1. RTs shorter than 150 ms or longer than 2,800 ms were removed from analyses. These amounted to 1.5% of the total number of trials. Error trials (9.8% of trials) were also removed. Figure 5 gives mean RTs and error rates in the two tasks (gender, emotion) across the experimental blocks (the baseline blocks were averaged). I first tested the presence of Garner interference, which records a difference in processing between filtering and baseline conditions. Mean RTs and accuracy rates were computed for each participants in the pertinent conditions. A two-way ANOVA with Task (gender, emotion) and Block (filtering, baseline) showed that neither of the effects of Task $\left[F_{\left(1,\;27\right)}=1.95,\;MSE=9120,\;\eta_{p}^{2}=0.067,\;p=0.17\right]$, Block $\left[F_{\left(1,\;27\right)}=2.53,\;MSE=4451,\;\eta_{p}^{2}=0.085,\;p=0.12\right]$, or their interaction [*F* < 1] was significant. A comparable analysis on error rates revealed that neither of the effects of Task $\left[F_{\left(1,\;27\right)}=1.97,\;MSE=0.0066,\;\eta_{p}^{2}=0.068,\;p=0.17\right]$, Block $\left[F_{\left(1,\;27\right)}=0.17,\;MSE=0.0001,\;\eta_{p}^{2}=0.0045,\;p=0.68\right]$, or their interaction $\left[F_{\left(1,\;27\right)}=3.41,\;MSE=0.0002,\;\eta_{p}^{2}=0.10,\;p=0.087\right]$ was significant. This entails that performance in filtering and baseline was on par. A set of complementary Bayesian ANOVAs on RTs and accuracy provided evidence for the absence of an effect (BF = 0.22, BF = 0.20, respectively). This outcome documents the absence of a Garner interference. Participants could pay perfect selective attention to the relevant dimension, while ignoring variations from the irrelevant dimension. According to this parity, the dimensions of gender and emotion are separable. This result is in accordance with those of Le Gal and Bruce (2002).

Next, I tested the influence of correlation on performance. Did observers reap gain due to the covariation of emotion and gender values? Such redundancy gains are often recorded as improved performance in the correlated block compared to the baseline block. Note however that the design consists of two types of correlated blocks (i.e., positive, negative). This requires two separate assessments. A two-way ANOVA with Task (gender, emotion) and Block (positive correlation, baseline) showed the effect of Block $\left[F_{\left(1,\;27\right)}=31.1,\;MSE=59,901,\;\eta_{p}^{2}=0.53,\;p<0.001\right]$, and its interaction with Task $\left[F_{\left(1,\;27\right)}=9.07,\;MSE=15,324,\;\eta_{p}^{2}=0.25,\;p<0.005\right]$ to be significant. Redundancy gains were found in both classification of emotion [*t*_(27)_ = 5.56, *p* < 0.001], and judgments of gender [*t*_(27)_ = 2.26, *p* < 0.05]. A similar analysis with the negatively correlated blocks revealed no effect whatsoever [*F* <]. Participants did not reap gain due to the negative covariation, possibly because the semantic conflict in this condition offsets the informational gain.

Next, I assessed the presence of the angry-men-happy-women bias in the data by looking at performance differences between the negatively and positively correlated blocks. A two-way ANOVA with Task (gender, emotion) and Block (positive correlation, negative correlation) showed the effect of Block $\left[F_{\left(1,\;27\right)}=21.1,\;MSE=69,180,\;\eta_{p}^{2}=0.43,\;p<0.001\right]$, and its interaction with Task $\left[F_{\left(1,\;27\right)}=12.47,\;MSE=36,933,\;\eta_{p}^{2}=0.31,\;p<0.005\right]$ to be significant. Positive correlation blocks (including only angry men and happy women) yielded faster performance than negative correlation blocks (consisting of only happy men and angry women). The effect of congruence in the correlation blocks was significant in judgments of emotion [*t*_(27)_ = 5.69, *p* < 0.001], but not in judgments of gender [*t* < 1]. Error data exhibited a significant main effect of Block $\left[F_{\left(1,\;27\right)}=7.56,\;MSE=0.03,\;\eta_{p}^{2}=0.21,\;p<0.05\right]$, suggesting that participants were more accurate in the positive than negative correlation blocks irrespective of task. These results support the presence of the angry-men-happy-women bias in the data as an across-blocks phenomenon.

### 9.1. Analyses at the Stimulus Level

**Figure 6:**
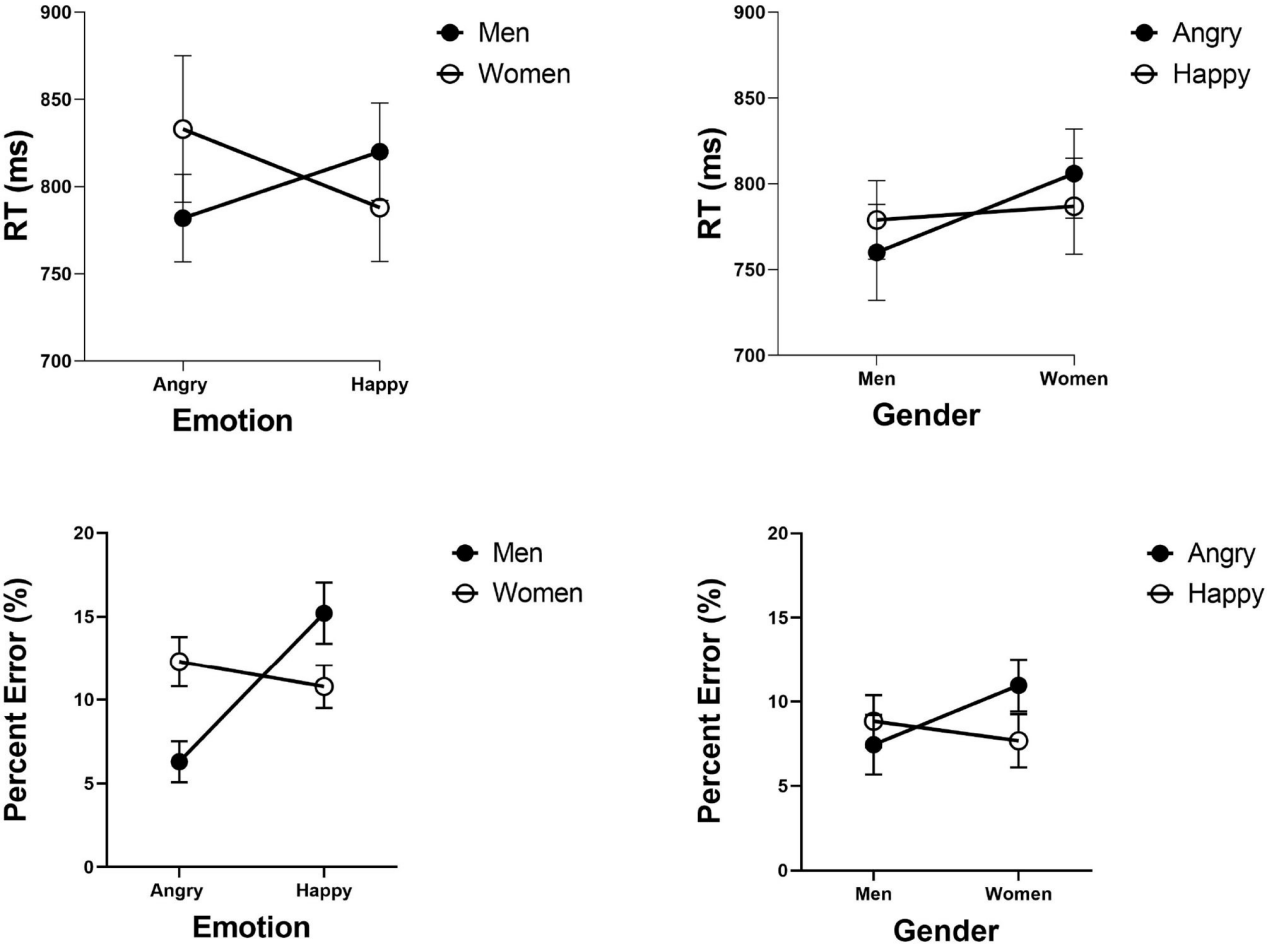
Experiment 1: Mean RTs and percent error (%) in categorization of emotion and gender as a function of emotion level (angry and happy) and gender level (men and women) in the filtering blocks of the emotion and gender tasks, separately. Error bars are standard error of the mean.

The judgmental bias also manifested itself as a within-block phenomenon in judgments of emotion. Figure 6 gives mean RTs and error rates in the filtering blocks for faces of angry men, angry women, happy men, and happy women, separately for judgments of emotion and gender. To assess the presences of the angry-men-happy-women confound in judgments of emotion, I coded the four types of face categories into two factors of emotion (angry, happy) and gender (male, female). ANOVA analyses on mean RTs showed their interaction to be highly significant [*F*_(1, 27)_ = 9.35, MSE = 46983, $\eta_{p}^{2}$ = 0.25, *p* < 0.005]. The main effects were not [*F* < 1]. Planned comparisons showed that angry men faces were responded faster than angry women faces [*t*_(27)_ = 2.60, *p* < 0.05]. The results for happy men and happy women were not significant [*t*_(27)_ = 1.44, *p* = 0.07]. A comparable analysis on error rates showed a similar pattern, with significant Gender x Emotion interaction $\left[F_{\left(1,\;27\right)}=11.4,MSE=0.038\;,\eta_{p}^{2}=0.29,\;p<0.005\right]$. More errors were committed with angry women faces than with angry men faces [*t*_(27)_ = 3.77, *p* < 0.005]. More errors were committed with happy men faces than with happy women faces [*t*_(27)_ = 2.03, *p* < 0.05]. These results replicate those by Becker et al. (2007) in their Study 2.

The same trend was numerically present in judgments of gender, but it was not supported by statistical analyses for both RTs $\left[F_{\left(1,\;27\right)}=1.49,MSE=9780,\eta_{p}^{2}=0.05,p=0.23\right]$, and error $\left[F_{\left(1,\;27\right)}=2.66,MSE=0.015,\eta_{p}^{2}=0.09,p=0.114\right]$. This outcome documents a failure to replicate the results of Study 3 in Becker et al. (2007). According to the current findings, the angry-men-happy-women is present only for judgments of emotion but not for classification of gender^4^.

## 10. Discussion

The results of Experiment 1 can be summarized as follows. First, at the dimensional level, facial emotion and gender appeared as separable dimensions. Neither of the dimensions produced Garner interference, suggesting that observers could pay perfect selective attention to the criterial dimension, while ignoring irrelevant variation on the unattended dimension. These results provide a conceptual replication of the findings by Le Gal and Bruce (2002). It should be noted though that gender and emotion did produce redundancy gains and stroop-like effects in the correlated blocks. These outcomes are consistent with integral processing (Algom and Fitousi, 2016). However, the outcome from the correlation blocks is often considered as weaker evidence for true perceptual integral processing because these blocks confound perceptual and decisional sources of interaction (Ashby and Maddox, 1994). Second, at the stimulus level, gender and emotion were not perceived independently. Specific combinations of stimulus values (men-angry, and women-happy) were processed more efficiently than others (men-happy and women-angry). This bias was evident in emotion categorization, but not in gender categorization. Therefore, these results provide only a partial replication of the findings by Becker et al. (2007).

The reader should note the conceptual distinction between (a) “dimensional level” interaction and (b) “stimulus level” interaction. The first type is measured across individual stimuli values. Garner interference is a prime example of such an interaction because it is measured as a difference in performance between two blocks, while ignoring the actual levels of individual stimuli (e.g., happy-men) in these blocks. The second type of interaction, “stimulus level” interaction is measured with respect to values of individual stimuli (e.g., angry-men), and therefore reflects cross-talk between values at the stimulus level. The angry-men-happy-women confound is a prime example of such an interaction. In the Garner paradigm we can measure this interaction by analyzing the responses to individual stimuli in the filtering blocks (see the section “Analyses at the stimulus level”). This distinction also exists in GRT. In GRT the “dimensional level” interaction is captured by violations of perceptual and decisional separability across stimuli, whereas the “stimulus level interaction” is captured by violations of perceptual independence.

Taken together, the outcomes from Experiment 1 pose a caveat. On the one hand, the dimensions appear as independent attributes, with no apparent cross-talk between them; on the other hand, when tested in the context of correlation, or at the item-level, the dimensions demonstrated strong interactions. This puzzle calls for further investigation using a more sophisticated approach to *perceptual independence* (Garner and Morton, 1969). Perceptual independence is not a unitary concept. There are several (not one) species of constructs, tasks, and measures of independence. This idea can be traced back to the seminal paper by Garner and Morton (1969), who provided the first rigorous treatment of perceptual independence and the need for converging operations (see also, Fitousi, 2013, 2015; Von Der Heide et al., 2018). Experiment 2 was designed to test for several types of perceptual independence and their possible violations using the GRT methodology.

## 11. Experiment 2

The aim of this experiment was to test the possible existence of perceptual and decisional interactions between facial emotion and gender. To this end, I applied the methodology known as GRT (Ashby and Townsend, 1986; Von Der Heide et al., 2018) with the same set of faces from Experiment 1. In contrast to the Garner speeded selective attention task, the GRT task is non-speeded divided attention task.

## 12. Method

### 12.1. Participants

Thirty young students from Ariel University took part (F = 27, M = 3, mean age = 22.86). All had normal or corrected-to-normal vision. All gave their signed consent. Non of these participants took part in Experiment 1. Note that different groups of participants were recruited for the Garner and GRT experiments. Ideally, it would be better to have the same participants performing the two tasks. However, the experiments were very demanding in terms of effort and time and there were some difficulties in recruiting participants during the COVID-19 for extended period of times.

### 12.2. Stimuli and Apparatus

The same set of faces from Experiment 1 was used. The faces were generated by a factorial intersection of the gender and emotion dimensions, yielding four type of faces: angry man, angry woman, happy man, and happy woman. The same facial identity could appear in either angry or happy expression. There were 10 faces of each combination, 40 faces in total (see Figure 4).

### 12.3. Procedure and Design

Participants were informed before the experiment about the four categories of faces in the experiment and how these categories were constructed. Participant were sitting in front of a laptop computer in a dimly lit room. On each trial a fixation cross was presented for 500 ms, and then was replaced by a blank screen for another 500 ms. Then a masking pattern appeared for 50 ms. The masking pattern was replaced by a single face that was chosen randomly by the computer and presented at the center of the screen for 40 ms. The face was then removed from the screen and a another masking pattern appeared for 50 ms. At this stage, participants categorized the presented face into one of four possible categories: (a) male-angry, (b) male-happy, (c) female-angry, and (d) female-happy. Participants respond with one of four response keys mapping: “m,” “n,” “x,” or “z.” Each key reflected a specific intersection of gender and emotion levels. The mapping between categories and keys was presented on the screen after each trial. No feedback was given. There were 15 experimental blocks. Each block consisted of 40 faces (see Figure 4) which were presented in random order. In total, each participant completed 600 trials. Participants were informed that the task is not speeded and that they should be as accurate as possible.

## 13. Results

### 13.1. Analyses of Accuracy

**Figure 7:**
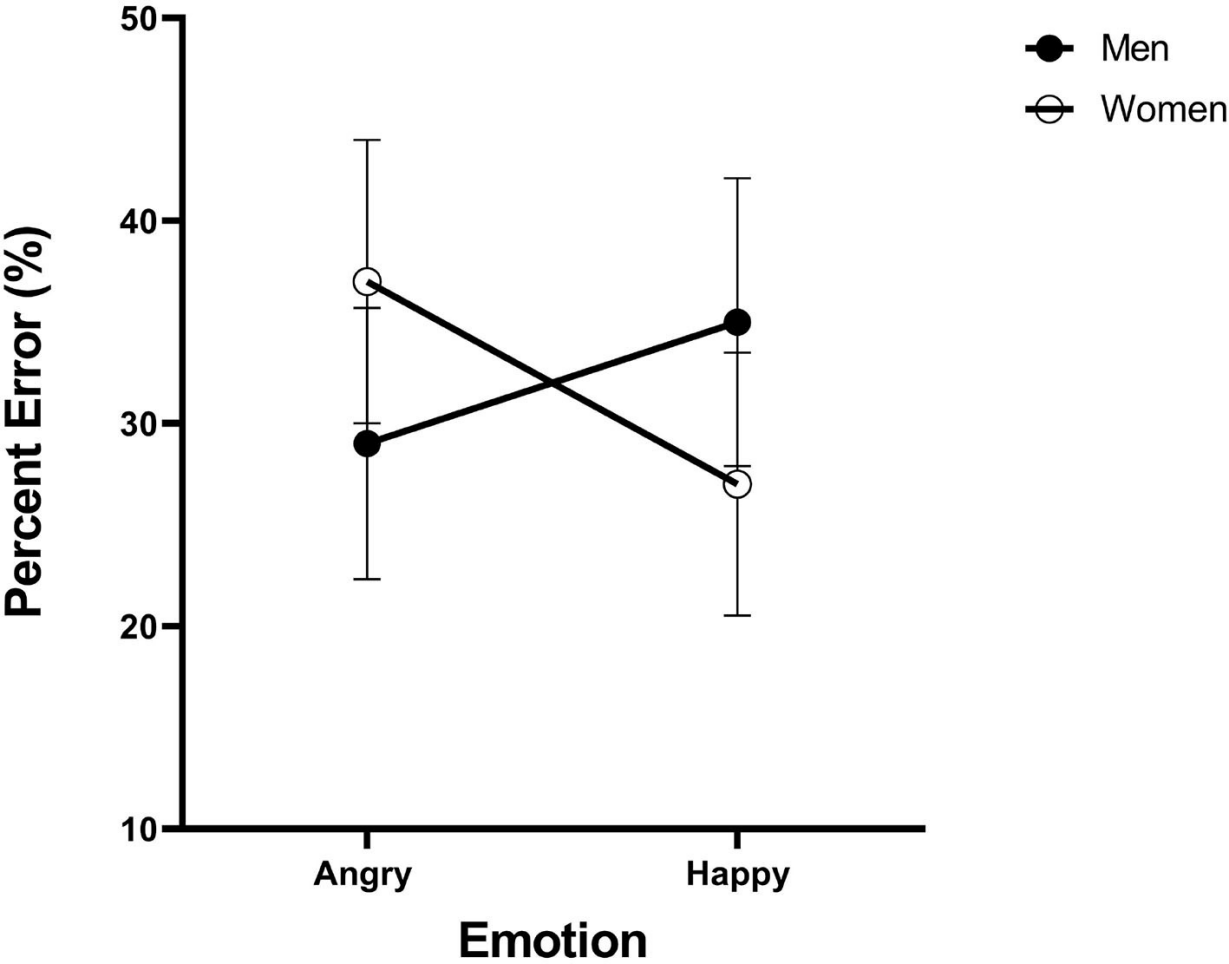
Experiments 2: Error rates (%) in the four emotion-gender intersections. Error bars are standard error of the mean.

Data can be downloaded from https://data.mendeley.com/datasets/zrz7krjhr4/1. Overall accuracy amounted to 67.6% correct. Figure 7 presents the mean error rates across participants for each type of face. As can be noted, the angry-men happy-women bias has surfaced in the accuracy data in full force. The stimuli were coded into two factors of emotion (angry, happy) and gender (male, female). ANOVA revealed that their interaction was highly significant $\left[F_{\left(1,\;23\right)}=29.3,MSE=0.19\;,\eta_{p}^{2}=0.50,\;p<0.005\right]$. Angry men faces were categorized more accurately than angry women faces [*t*_(29)_ = 2.84, *p* < 0.005], and happy women faces were categorized more accurately than angry women faces [*t*_(29)_ = 2.48, *p* < 0.005]. These results document the angry-men-happy-women bias in the accuracy rates. These results extend earlier findings from speeded selective attention tasks to the non-speeded divided attention task of complete identification. Next, GRT analyses will assist us in discovering what representational states generate these accuracy patterns.

### 13.2. GRT-wIND Analyses

There are several approaches to making inferences regarding the structure of GRT spaces and the associated constructs of perceptual independence, perceptual separability and decisional separability. Ashby and Townsend (1986) developed a battery of non-parametric tests for assessing failures of these constructs (see also, Thomas, 2001). Kadlec and Townsend (1992) and Kadlec (1995) have advanced a set of micro- and macro- signal detection analyses aiming at the same goal. Ashby and Perrin (1988) and Ashby (1992) advanced a model-based approach to GRT that consisted of fitting parametric models to data under the assumption that the distributions are Gaussian (Thomas, 2001; Fitousi, 2014, 2018). These approaches proved valuable (Richler et al., 2008), but when these approaches are applied to the popular 2 x 2 design, two major problems may arise (Soto et al., 2015, 2017): (a) the number of free parameters is greater than the number of data points, and therefore some assumptions about parameters, and therefore about constructs, should be made, and most importantly (b) violations of perceptual independence can be mimicked by violations of decisional separability, and vice versa (Silbert and Thomas, 2013). The latter issue may pose a real threat to the validity of GRT (Mack et al., 2011). However, a recent development by Soto et al. (2015) has solved these issues in a rigorous and creative way.

Soto et al. (2015, 2017) have developed the General Recognition Theory with Individual Differences (GRT-wIND) model^5^. The GRT-wIND assumes that perceptual separability and perceptual independence either hold or fail across all participants, and that all participants perceive the set of stimuli in a similar fashion. Other aspects of the model such as decisional separability and weighting of attention to the dimensions, vary across observers and reflect individual differences. The model is fit simultaneously to all the individual observers' data. The GRT-wIND is the only available way of dissociating perceptual and decisional components in the 2 x 2 design. This approach is deployed here to disentangle perceptual and decisional contributions to the interaction between gender and emotion.

**Figure 8:**
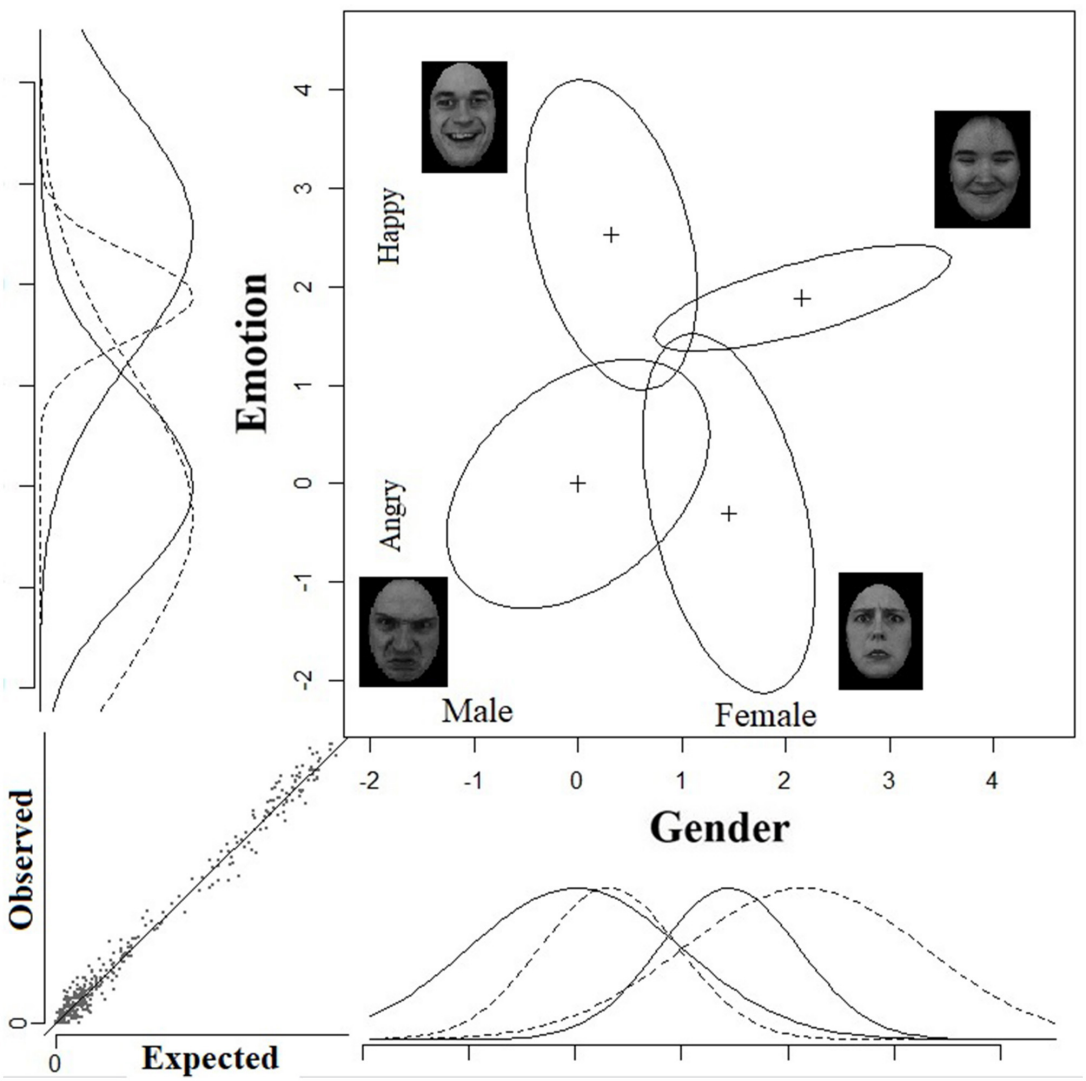
Experiment 2: Best fitting GRT-Wind space. The unidimensional distributions on the *x*-axis with the solid lines represent the effect of gender for the two lower stimuli, whereas the unidimensional distributions with the dashed lines represent the perceptual effect of gender for the two upper stimuli. Analogs unidimensional distributions on the *y*-axis represent the perceptual effect of emotion for the corresponding stimuli. The small graph on the bottom left corner illustrates the relations between observed and predicted probabilities for each of the matrix entries for each observer.

I have used the GRT-wIND package (Soto et al., 2015, 2017) in the open source software R (R Core Team, 2017) to analyze the data. Identification confusion matrices were derived for each observer. These matrices were entered into the software for further analysis. Table 2 gives the results of the GRT-wIND fitting. The best fitting model (*log likelihood* = −12105.97, *R*^2^ = 0.987) was a full model with violations of perceptual separability on Gender [χ^2^_(4)_ = 41.0, *p* < 0.001], violations of perceptual separability on Emotion [χ^2^_(4)_ = 62.47, *p* < 0.001], violation of decisional separability on Gender [χ^2^_(4)_ = 211.50, *p* < 0.001], violation of decisional separabiilty on Emotion [χ^2^_(4)_ = 128.46, *p* < 0.001], and violations of perceptual independence [χ^2^_(4)_ = 50.55, *p* < 0.001]. Figure 8 illustrates the GRT-wIND space for this best-fitting model. As can be noted, the equal-likelihood contours represents the joint effects of emotion and gender for each of the four type of face categories (angry-men, angry-women, happy-men, and happy-women).

**Table 2:** Experiment 2: Results of the best fitting GRT-wIND model.

**Test**	**χ^2^**	**DF**	***p*** **-value**	**Violation**
Perceptual separability of gender	41.0	4	<0.001	YES
Perceptual separability of emotion	62.47	4	<0.001	YES
Perceptual independence	50.55	4	<0.001	YES
Decisional separability of gender	211.50	24	<0.001	YES
Decisional separability of emotion	128.46	24	<0.001	YES

The spatial configuration of the contours and their shape can tell us much about the form of interactions between the dimensions. It can be noted that perceptual separability of gender is violated because the discriminability of gender changes as a function of emotion level. Male and female faces are more discriminable when they are happy than when they are angry. Similarly, perceptual separability of emotion is violated, because the discriminability of emotion is altered when gender levels are changed from male to female. Violations of perceptual separability entail that the dimensions are not independent.

Most importantly, violations of perceptual independence—a form of within stimulus independence—are highly discernible in all four face categories. Perceptual independence is violated for each of the four types of emotion-gender intersections. The form of violation is consistent with the direction of the social bias for the pertinent type of intersection. As can be noted, the contours appear as tilted ellipses—a sure sign for violations of perceptual independence. The direction of the tilt is determined by the correlation coefficient ρ parameter in the model. The correlation coefficient is positive for angry-men (ρ = +0.40) and happy-women faces (ρ = +0.72), and negative for the happy-men (ρ = −0.36) and angry-women (ρ = −0.41) faces. This result is remarkable because perceptual independence (or its violations thereof) is a form of within-stimulus independence. The fact that it is violated in all four face categories, and particularly according to the hypothesized confound observed in other empirical studies, suggests that the confound has strong perceptual sources, and that it is deeply entrenched. The positive correlation in the “angry-male” distribution, for example, entails that as the face is perceived as more masculine it is also perceived as more angry and vice versa. The negative correlation in the “happy-male” distribution entails that as this type of faces generate a more masculine perceptual effect they are perceived as less happy and vice versa.

Finally, decisional separability has been also found to be violated on both gender and emotion. The GRT-wIND identifies for each participant his or her specific decision thresholds, these cannot be presented in Figure 8. The point to note is that the best-fitting model incorporates violations of decisional separability, which means that the decision criteria for gender categorization changes as a function of emotion levels, and similarly, the decision criteria for emotion categorization is not the same at the two gender levels. One obvious limitation of the GRT-wIND approach is that it does not provide the estimates of the averaged decision bounds across participants on the two dimensions, as it does with respect to perceptual independence and perceptual separability. This is due to the main assumption in GRT-wIND that decision bounds vary across participants. As a result, we cannot relate directly the placement of the decision bounds on the GRT spaces to the generation of the angry-men-happy-women bias. But we can safely argue that there are decisional biases in judgments of emotion and gender and that these dimensions interact at a decisional level (in addition to a perceptual level). We cannot conclusively argue that these decisional bounds generated the bias observed at the level of mean accuracy.

## 14. Discussion

The GRT analyses provide strong evidence that the facial dimensions of emotion and gender are not independent dimensions. Moreover, not one but several varieties of independence are violated. These included interactions at the decisional level (violations of decisional separablity) and at the perceptual level (violations of perceptual separability). Most importantly, violations of perceptual independence—a form of within-stimulus independence—revealed that the cross-talk between the values of the dimensions within a given category is strong and consistent with the direction of the angry-men-happy-women bias observed in the current Garner study (Experiment 1) and previous studies who deployed speeded tasks. Congruent stimuli (angry-men and happy-women) exhibited positive correlation coefficients that governed their internal representation (the equal likelihood contours), whereas incongruent stimuli (happy-men and angry-women) showed negative correlation coefficients underlying their internal representations.

It seems that the GRT results are not fully consistent with the Garner results. They are in line with the findings of redundancy gains, but not with the absence of Garner interference. One would expect that the absence of Garner interference, which signals dimensional separability, should also imply perceptual and decisional separability in the GRT. This point will be elaborated in the General Discussion, but for now suffice it to say that the degree to which GRT and Garner task constructs align with each other is not well understood (see for, Fitousi and Wenger, 2013; Algom and Fitousi, 2016).

## 15. Experiment 3

The goal of Experiment 3 was to generalize the results of Experiment 2 with another set of stimuli. Experiment 2 tested the intersection of angry/happy and male/female values. However, Le Gal and Bruce (2002) have used a slightly different composition, with levels of anger/surprise and male/female. In this setting, the dimensions also produced no Garner interference, and yielded a social bias (i.e., angry-men surprised-women bias). In particular, faces of angry-men and surprised-women were processed more efficiently than faces of angry-women and surprised-men. It is important to show, however, that the GRT patterns observed in Experiment 2 can be replicated with the angry/surprise type of faces.

## 16. Method

### 16.1. Participants

Twenty four young students from Ariel University took part (2 man and 22 woman). Their mean age was 23.08 years. All had normal or corrected-to-normal vision. All gave their signed consent.

### 16.2. Stimuli and Apparatus

**Figure 9:**
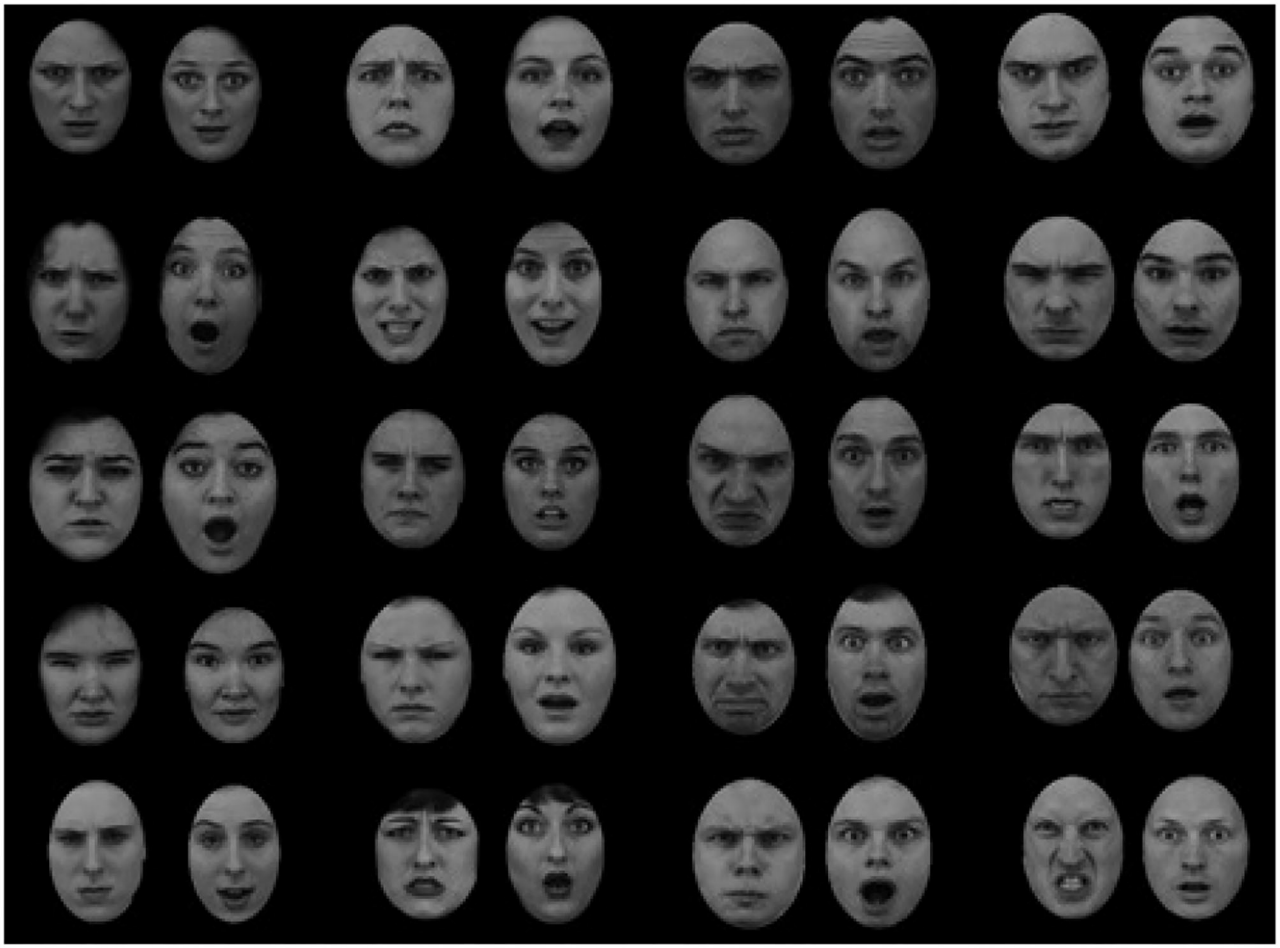
Experiment 3: The faces were created by factorial combination of two levels of emotion (angry and surprised) with two levels of gender (male and female). The face images were taken with permission from the Karolinska Directed Emotional Face (KDEF, Lundqvist et al., 1998).

The face images were taken with permission from the Karolinska Directed Emotional Face (KDEF, Lundqvist et al., 1998). The images were standardize by transforming them into gray-scale images, cropping the hair of each face and placing it within a standard oval shape of approximately 7 × 9 cm viewed from an approximate distance of 56 cm. The faces varied on gender (male, female) and emotion (angry, surprise). There were four face categories: angry man, angry woman, surprised man, and surprised woman. The same facial identity could appear in either angry or surprised expression. There were 10 faces of each combination, 40 faces in total (see Figure 9).

### 16.3. Procedure and Design

These were identical to those reported in Experiment 2.

## 17. Results

### 17.1. Analyses of Accuracy

**Figure 10:**
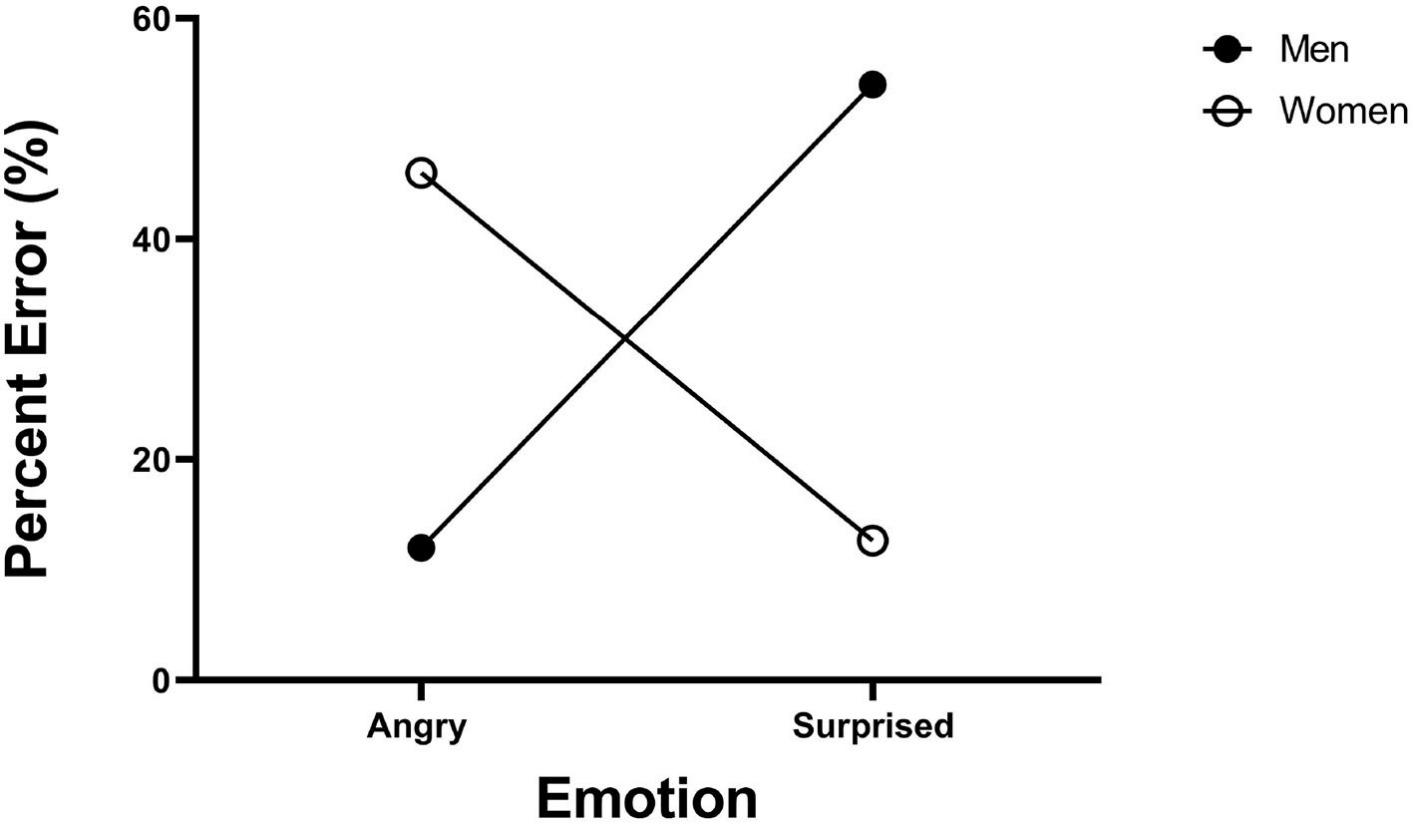
Experiment 3: Error rates (%) across the four emotion-gender categories. Error bars were too small to be shown.

Data can be downloaded from https://data.mendeley.com/datasets/zrz7krjhr4/1. Overall accuracy in the complete identification task amounted to 68.4% correct. Figure 10 gives the mean error rates for each type of face category. A visual inspection reveals a strong bias in categorization. The stimuli were coded into two factors of emotion (angry, surprised) and gender (male, female). ANOVA revealed that the interaction was highly significant $\left[F_{\left(1,\;23\right)}=107.8,MSE=3.53\;,\eta_{p}^{2}=0.82,\;p<0.005\right]$. Angry men faces were categorized more accurately than angry women faces [*t*_(23)_ = 6.68, *p* < 0.001], and surprised women faces were categorized more accurately than surprised men faces [*t*_(23)_ = 9.68, *p* < 0.005]. These results replicate the earlier findings of the angry-man-surprised-woman bias observed by Le Gal and Bruce (2002).

### 17.2. GRT-wIND Analyses

**Figure 11:**
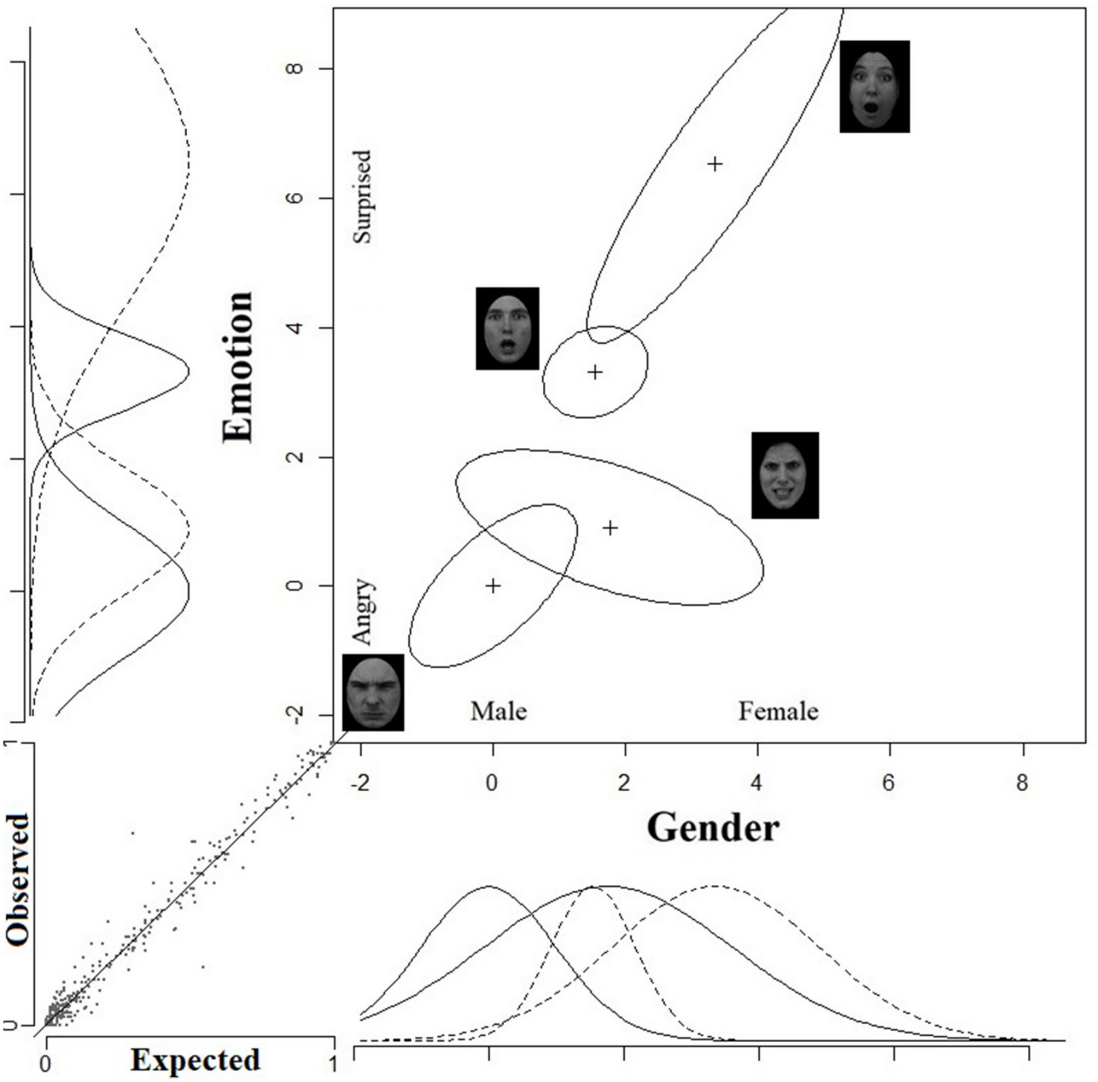
Experiment 3: Best fitting GRT-Wind space. The unidimensional distributions on the *x*-axis with the solid lines represent the effect of gender for the two lower stimuli, whereas the unidimensional distributions with the dashed lines represent the perceptual effect of gender for the two upper stimuli. Analogous unidimensional distributions on the *y*-axis represent the perceptual effect of emotion for the corresponding stimuli. The small graph on the bottom left corner illustrates the relations between observed and predicted probabilities for each of the matrix entries for each observer.

Identification confusion matrices were derived for each observer. These matrices were subjected to analysis using the R GRT-wIND package (Soto et al., 2015, 2017). Table 3 present the results of the GRT-wIND fitting procedure. The best fitting model (*log likelihood* = −99, 332, *R*^2^ = 0.978) was a full model with violations of perceptual separability on Gender [χ^2^_(4)_ = 70.9, *p* < 0.001], violations of perceptual separability on Emotion [χ^2^_(4)_ = 231.2, *p* < 0.001], violation of decisional separability on Gender [χ^2^_(4)_ = 1902.8, *p* < 0.001], violation of decisional separabiilty on Emotion [χ^2^_(4)_ = 344.4, *p* < 0.001], and violations of perceptual independence [χ^2^(4) = 221.2, *p* < 0.001]. A graphical illustration of the best-fitting GRT-wIND space is presented in Figure 11.

**Table 3:** Experiment 3: Results of the best fitting GRT-wIND model.

**Test**	**χ^2^**	**DF**	***p*** **-value**	**Violation**
Perceptual separability of gender	70.9	4	<0.001	YES
Perceptual separability of emotion	231.2	4	<0.001	YES
Perceptual independence	221.2	4	<0.001	YES
Decisional separability of gender	1902.8	24	<0.001	YES
Decisional separability of emotion	344.4	24	<0.001	YES

As can be noted in Figure 11, perceptual separability for gender is violated. The horizontal distance between the distributions that represent the face categories of surprised men and surprised women is larger than the horizontal distance separating the comparable distributions for angry men and angry-women. Similarly, perceptual separability of emotion is violated. The distance (=discriminability) separating the distributions of angry women and surprised women is larger than that separating the distributions of angry men and surprised men. Most importantly, there were glaring violations of perceptual independence in all four face categories. The equal contours appear as tilted ellipses, indicating violations of perceptual independence within each of the individual stimuli (or face categories). The direction of the tilt is determined by the correlation coefficient ρ parameter in the model. The correlation coefficient values for each contour entail large positive correlations for the angry-men (ρ = +0.65) and surprised-women faces (ρ = +0.89), a small and positive correlation for surprised-men faces (ρ = +0.20), and a large and negative correlation for angry-women faces (ρ = −0.55). This pattern provides almost a full replication of the results of Experiment 2. The only inconsistency is in the surprised-men distribution which should have shown a negative correlation instead of the small positive correlation. However, overall the dramatic violations of perceptual independence and their direction are consistent with the hypothesis that the angry-man-surprised-woman bias is contributed by a strong perceptual component.

Finally, decisional separability has been also found to be violated on both gender and emotion. Because GRT-wIND identifies for each participant his or her specific decision bounds, these cannot be presented in Figure 11. But the model identifies highly significant changes in the placement of decision bounds for each dimension as a function of levels on the other dimension. These results strongly suggest that emotion and gender do not interact at a purely perceptual level, but also at a decisional level. These outcomes support the conclusion that emotion and gender interact at multiple representational levels. However, one should be cautious in claiming that these decisional biases generated the angry-men-happy-women bias because the GRT-wIND does not provide an estimate of the average decision bounds across participants, only the individual values.

## 18. General Discussion

Previous studies (Le Gal and Bruce, 2002; Becker et al., 2007) have documented a glaring confound between facial emotion and gender. Facial expressions of anger were found to be associated with male faces, while facial expressions of happiness/surprise were found to be associated with female faces. Two primary theoretical alternatives have been proposed to account for this bias. According to a top-down stereotypical account, the bias is due to the impact of well-learned or imagined associations between levels of emotion and gender^6^. According to a bottom up (perceptual) account, the bias is the result of a built-in confound between morphological cues for emotion and gender. The two alternatives have proved difficult to tear a part, mainly because the experimental methodologies that were used confounded perceptual and decisional biases. These methodologies also fell short of distinguishing between interactions at the stimulus and dimensional levels. In particular, the dimensions of emotion and gender were found to be separable in the Garner paradigm, an outcome that fly in the face of the interaction found at the stimulus level. Moreover, the Garner paradigm cannot dissociate perceptual and decisional types of interactions. Therefore, the concept of independence (or interaction) as gauged in these methodologies needed a principled reexamination. To accomplish this goal, the present study harnessed the GRT, in addition to the Garner paradigm.

Experiment 1 applied the Garner paradigm to happy and angry faces of women and men. The dimensions were found to be separable, but the angry-men happy-women bias surfaced in both the correlated blocks and the individual face categories. These results replicate and extend earlier findings (Le Gal and Bruce, 2002; Becker et al., 2007). Experiment 2 applied the GRT to the same set of stimuli from Experiment 1. This time, the dimensions were found to be dependent. A within-stimulus form of interaction—violation of perceptual independence—has been observed in all four type of face categories (angry men, happy men, angry women, happy women). The sign of the within-stimulus correlation was consistent with the type of face category, such that positive correlations were found in the angry-men and happy-women categories, and negative correlations were observed in the angry-women and happy-men categories. This outcome indicates that the bias is generated by bottom-up processes. In addition, violations of perceptual and decisional separability were observed on both dimensions. Violations of perceptual separability, like violations of perceptual independence, reflect the contributions of bottom-up components, whereas violations of decisional separability entail strategic top-down components (e.g., stereotypes). Experiment 3, extended these conclusions to faces with angry and surprised emotional expressions^7^.

### 18.1. Varieties of Biases in the Processing of Emotional Faces

Becker et al. (2007) have argued that the interaction between gender and emotion is perceptual in nature: “We thus believe that parsimony favors an explanation at the level of signal itself–that there is a natural confound between the features that correspond to maleness and anger, on the one hand, and femaleness and happiness, on the other. Nonetheless, it remains a possibility that social learning processes may also play a role in creating these phenomena.” (p. 189). These authors posit a distinction between perceptual and decisional sources of interaction and relate them to bottom up and top-down sources, a distinction that is accepted by many students of face perception (Johnson et al., 2012), and is pursued in the current study. The present study is consistent with both bottom-up (perceptual) and top-down (decisional) influences, as it shows that stereotypical processing of emotional faces involves several perceptual and decisional interactions. The interactions occur at various levels of representation: (a) perceptual interactions at the level of the individual face, (b) perceptual interactions at the dimensional level, and (c) decisional interactions at a response level. These results are consistent with the idea that the dimensions are largely integral and that the interaction takes place at several, not one, representational loci. It should be acknowledged though that the distinction between bottom-up and top-down processes has been recently criticized for being inadequate in the attention literature (Awh et al., 2012). However, it is still considered as immensely instrumental by others (Theeuwes, 2010). The dichotomy continues to affect major theories in the areas of social cognition (Freeman and Ambady, 2011), categorization (Ashby and Soto, 2015), and face recognition (Wegner and Ingvalson, 2002).

A limitation of the current study should be acknowledged with respect to its ability to exactly specify whether and how violations of decision separability generate the angry-men-happy-women bias. Our choice of using the GRT-wIND was based on the fact that it is the only available tool that can dissociate perceptual and decisional interactions in a 2 × 2 GRT designs (Soto et al., 2017). GRT-wIND succeeds in circumventing methodological problems in GRT (Silbert and Thomas, 2013, 2014) by assuming that the exact placements of decisional bounds vary across participants. However, this success comes with a price tag. The GRT-wIND does not provide the average estimate of bounds across participants. As a result, we could not directly relate the placement of the decision bounds on the GRT spaces to the generation of the angry-men-happy-women bias at the group level. We can, however, safely conclude that emotion and gender interact at a decisional (in addition to a perceptual) level, but we cannot conclusively argue that these decisional interactions generated the angry-men-happy-women bias. However, it is highly likely that for most participants this is the case.

Another comment is in order regarding the relations between the Garner and GRT results. If the dimensions indeed exhibited violations of perceptual separability and independence in the GRT, how come they have not produced Garner interference? This finding is particularly challenging to the Garnerian edifice, since the Garner and GRT paradigms have been often considered as converging operations (Ashby and Townsend, 1986; Fitousi and Wenger, 2013; Algom and Fitousi, 2016). The answer to this caveat is that the relations between those approaches are not as clear cut as has been assumed (Ashby and Maddox, 1994). First, we still do not know whether Garner interference implies violations of decisional or/and perceptual separability and whether the opposite holds true. Moreover, The Garner paradigm is not designed to dissociate perceptual and decisional separability, nor to record violations of perceptual independence at the stimulus level (Soto et al., 2017). Those can only be measured in the GRT. Second, although they go by the same name, the constructs of perceptual separability in GRT and Garner might not correspond to each other (Algom and Fitousi, 2016). When tested empirically with facial identity and emotion, Fitousi and Wenger (2013) have recorded only a medium level of correspondence between the two. The upshot is that the psychological concept of independence is a nomenclature that can refer to various measures, definitions, and tasks, not all of them necessarily converge on the same meaning (Garner and Morton, 1969).

### 18.2. Implications for Models of Face Recognition

It is interesting to consider the current findings in the context of dual-route models of face recognition (Bruce and Young, 1986; Haxby et al., 2000). According to the model by Haxby et al. (2000) separate neural systems are involved in recognition of faces. One system is dedicated to the processing of invariant aspects of faces (e.g., identity, gender) and another to the processing of variant aspects of faces (e.g., emotion, lip movements). This functional dichotomy entails independence of variant and invariant attributes (e.g., emotion and identity), a prediction that has not been corroborated by the current study. Emotion which is a variant aspect of faces and gender which is an invariant attribute of faces interacted at several perceptual and decisional loci. Fitousi and Wenger (2013) have reached similar conclusions with respect to the dimensions of emotion and identity. The logic guiding that study was similar to the present one, applying three different methodologies (including the Garner and GRT paradigms) to assess independence. These results cast doubts on the strict dichotomy postulated by dual-route models between various sources of information^8^.

### 18.3. Implications for Other Aspects of Social Categorization and Bias

Although the current study focuses exclusively on how gender affects emotion categorization, I suggest that similar perceptual and decisional biases will exist for other social categorization. Recent years have seen a growing interest in social biases in judgments of facial dimensions. In many respects, speeded and non-speeded face categorization tasks (Oosterhof and Todorov, 2008; Freeman et al., 2012) have replaced traditional paper and pencil tasks (Fiske and Neuberg, 1990) in pinpointing the locus of social biases. Social biases are now detected in experiments in which face categories are intersected and speeded or non-speeded responses to these faces are recorded (Hugenberg and Bodenhausen, 2003; Fitousi, 2020). An influential study by Johnson et al. (2012) demonstrated how categorization of faces by race can be affected by the gender of the face. Johnson et al. (2012) proposed that such biases may be widespread, and influence the earliest stages of social perception, categorizations. They have proposed that race categories may bias gender categorization via two routes–one resulting from shared facial cues, and the other emerging through shared social stereotypes. Other studies (Schweinberger et al., 2010; Wiese et al., 2013; Fitousi, 2020) have shown that judgments of age are biased by the gender of the face. An important theoretical distinction that is common to all of these studies concerns the relative contributions of bottom-up and top-down processes. Dissociating between these two types of processes has proven extremely difficult.

In their *dynamic interactive theory* of person construal Freeman and Ambady (2011) have modeled these processes within the framework of a dynamical system that involves continuous interaction between social categories, low-level processing, high-level cognitive states and stereotypes. Person construal is determined by the accumulation of activation from various nodes at various representational levels (e.g., low-level features, high-level concepts, stereotypes). In this model bottom-up and top-down processes operate in parallel, moving the system into its stable state. Another model that can account for bias in person construal is the “face file” approach (Fitousi, 2017a,b). In this framework social categories of faces (e.g., emotion, gender) are represented as episodic events (Hommel, 2004) across space and time. The features of these categories (e.g., angry, female) are subjected to a binding process. The bindings is facilitated if the dimensions share common spatial or motor codes. This framework can explain biases in categorization of emotional faces and other social categories as a tendency to bind related perceptual and motor codes, extending the scope of interactions to the realm of action and affordances (Gibson, 1979).

One issue that deserves a comment concerns the role of awareness in categorization of face dimensions. One may ask to what extent were the participants aware of the dimensional levels of gender and emotion in the present study? The Garner paradigm has been designed to test for selective attention and not consciousnesses, and we note that awareness and attention are two dissociable constructs (Lamme, 2003), albeit their relatedness. In the Garner paradigm, participants likely attended to the two dimensions in the correlated blocks, because redundancy gains have been observed. As for the filtering blocks, I cannot determine exactly whether participants were aware of the irrelevant dimension levels. However, the findings of the angry-men-happy-women interaction may indicate that participants processed the irrelevant dimension to some degree. The absence of Garner interference along with interactive effects at the stimulus level can be accounted for by a two-stage model of perception, according to which the dimensions are initially processed as dependent dimensions, but then become independent (Fitousi, 2020). In the GRT complete identification task, both dimensions were available to the observers' consciousness because the task required divided attention.

The questions of awareness and dimensional interaction can be investigated in a rigorous fashion by using adaptation methods (McCollough, 1965). The *contingent adaptation* technique has been applied to gender and eye distance (Little et al., 2005), gender and ethnicity (Ng et al., 2006), and gender and emotion (Harris and Ciaramitaro, 2016). In this method, researchers look for evidence of contrasting aftereffects based on specific feature combinations (e.g., angry male and happy female). If the two features are independent, there should be no net adaption, since there is an equal amount of exposure to opposing conditions. If, on the other hand, opposing aftereffects do emerge, then evidence is adduced that the features are dependent. This is exactly what the study by Harris and Ciaramitaro (2016) has aimed at testing. In Experiment 1 of this study, participants were adapted to angry female and happy male faces. After adaption, female faces were judged as happier than the PSE (point of subjective equality), and male faces were judged as angrier than at the PSE. These contrastive aftereffects are consistent with integralilty of emotion and gender. However, in Experiment 2 in which participants were adapted to angry male and happy female faces, these authors failed to provide evidence for such contrastive aftereffects. The results from the contingent adaptation technique are not conclusive, but they can at least give some support to the idea that gender and emotion interact at a perceptual level, and be extracted without attention or awareness.

## Data Availability Statement

Data can be downloaded from https://data.mendeley.com/datasets/zrz7krjhr4/1.

## Ethics Statement

The studies involving human participants were reviewed and approved by Ariel University Ethical Committee. The patients/participants provided their written informed consent to participate in this study.

## Author Contributions

The author confirms being the sole contributor of this work and has approved it for publication.

## Funding

This research was supported by the ISRAEL SCIENCE FOUNDATION (grant no. 1498/21).

## Conflict of Interest

The author declares that the research was conducted in the absence of any commercial or financial relationships that could be construed as a potential conflict of interest.

## Publisher's Note

All claims expressed in this article are solely those of the authors and do not necessarily represent those of their affiliated organizations, or those of the publisher, the editors and the reviewers. Any product that may be evaluated in this article, or claim that may be made by its manufacturer, is not guaranteed or endorsed by the publisher.

## Footnotes

^1^There is the possibility that both top-down (decisional) and bottom-up (perceptual) biases are involved in social judgment of gender and emotion (Freeman and Ambady, 2011).

^2^For GRT in non-Cartesian spaces see Townsend and Spencer-Smith (2004).

^3^The distinction between bottom-up and top-down processes is a cornerstone of the dynamic-interactive model by Freeman and Ambady (2011). This model has been developed to account for a broad class of phenomena in face categorization, including the angry-man-happy-woman bias. These researchers have used a dynamic system to account for the influence of featural overlap at the physical level (perceptual factors) and learned stereotypes (top-down) on the absolute time of face categorization.

^4^I tested the possibility that the order of tasks (e.g., gender, emotion) may have influenced the strength of the angry-male association, leading to weaker association when the gender task is performed first. This conjecture was not supported by statistical analysis as the three-way interaction of Order x Emotion x Gender was not significant [*F* < 1].

^5^I note that Soto et al. (2017) have also implemented a so called “Garner test” in their GRT-wIND package. However, while their approach to GRT is innovative and useful, the procedures advanced by Soto et al. (2017) under the tag “Garner paradigm” are problematic. In a matter of fact, these advances represent a considerable deviation from the original intent of Garner and his students (Algom and Fitousi, 2016). First, the Garner design presented by Soto et al. (2017) uses only the filtering block, while neglecting the essential baseline and correlated blocks. This is very unusual because the Garner test relies on computing differences in performance across blocks. Second, Soto et al. (2017) have replaced the original Garner interference test with the marginal Response Time invariance (mRTi). This test is conceptually different from the Garner interference or redundancy gains tests. While the traditional Garner tests look at differences at the level of the mean RTs and mean accuracy across experimental blocks, the mRTi compares entire RT distributional shapes within the filtering block. Third, the mRTi test itself relies on an uncertain assumption—the RT-distance assumption. According to this assumption response times become faster as the internal percept is farther from the decision bound. Nosofsky and Palmeri (1997) have adduced strong evidence against the RT-distance assumption. The upshot is that the Soto et al. (2017) test is not the original Garner paradigm albeit the common name, and that it requires theoretical and distributional assumptions that are not required in the original paradigm.

^6^One reviewer rightly noted that statistical learning effects may affect the perceptual rather than decisional level.

^7^Most participants in the three experiments were females. I could not refute the logical possibility that the angry-men-happy-women bias is stronger in female than in male participants due to the scarcity of male participants. Previous studies who have tested for this possibility (Becker et al., 2007), have not found any evidence for it.

^8^An interesting possibility raised by the current results is that dimensional interactions between variant facial dimensions, such as emotional expression and gaze direction (Pecchinenda et al., 2008; Pecchinenda and Petrucci, 2016, 2021) exert interactive effects only under certain conditions that require an affective evaluation, or when the two dimensions have a shared mechanism (Adams and Kleck, 2005).
